# Supplementary material for: Development of a novel-ionizable-lipid-based mRNA vaccine for broad protection against Streptococcus pneumoniae
Source: Mol Ther Nucleic Acids. 2025 Sep 1;36(4):102699. doi: 10.1016/j.omtn.2025.102699 (PMC12744836; doi:10.1016/j.omtn.2025.102699)
Supplement: Document S1. Figures S1–S5 [file mmc1.pdf]

OMTN, Volume *36*

Supplemental information

Development of a novel-ionizable-lipid-based mRNA vaccine for broad protection against *Streptococcus pneumoniae*

Shi Xu, Guoqing Qi, Rui Liu, Shang Liu, Aili Wang, Wei Li, Keyue Ruan, Lingzhi Zhan, Lianshun Wang, Caiyi Fei, Jiyang Zhao, Xue Zhang, Qin Yu, Mengwei Xu, Jing Li, and Tiyun Han

Supplemental Information

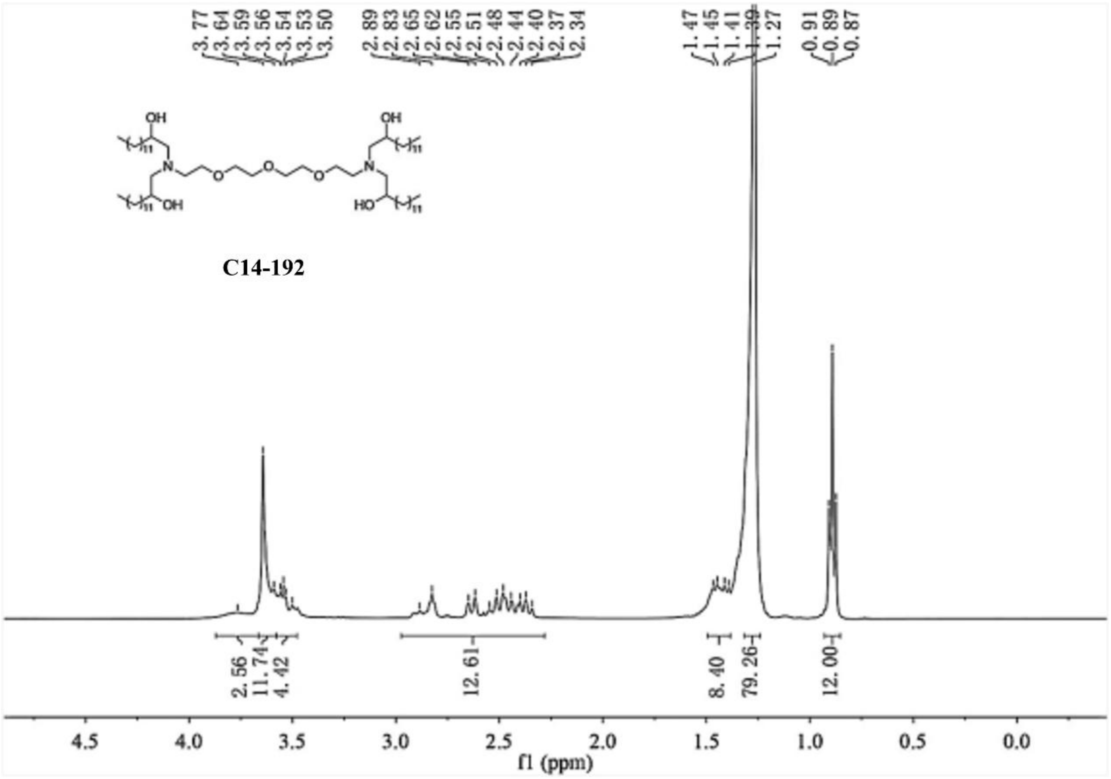

Figure S1. 1H nuclear magnetic resonance (1H NMR) spectra analysis of synthesized C14-192 cationic lipid compound.

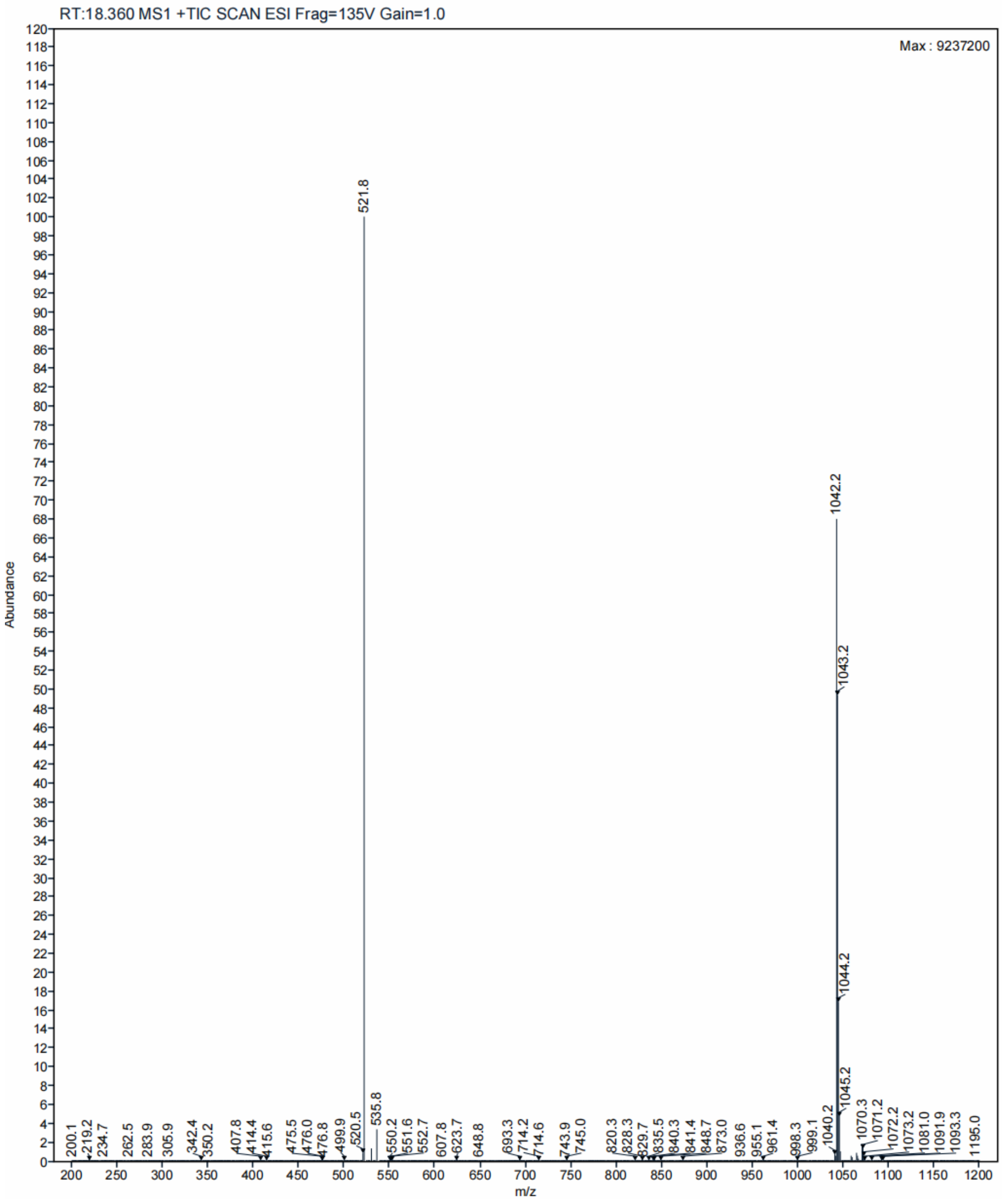

Figure S2. Liquid chromatography-mass spectrometry (LC-MS) analysis of synthesized C14-192 cationic lipid compound.

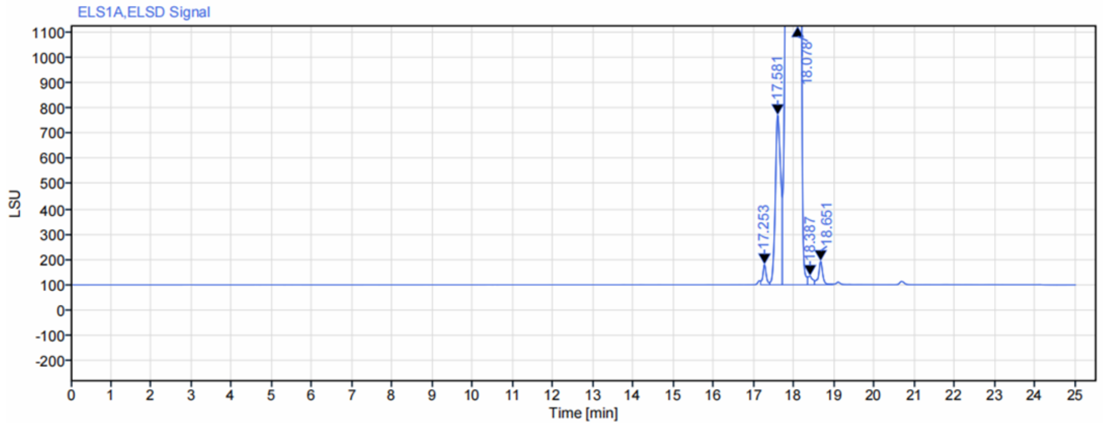

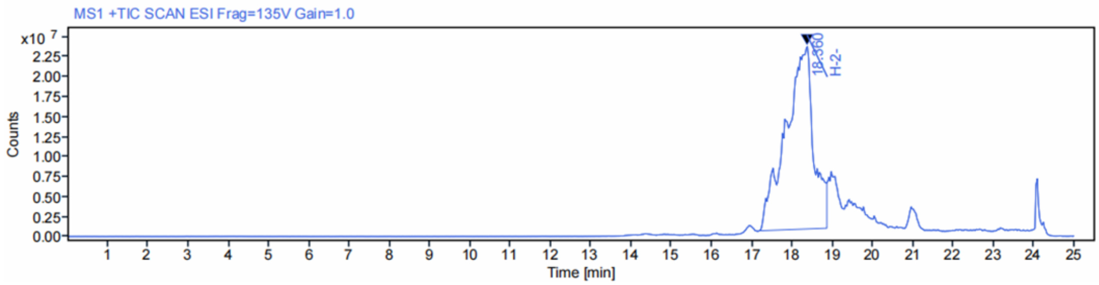

Signal: ELS1A,ELSD Signal

Name	RT [min]	RRT	Area	Height	Area%	TF	Plates	S/N	Rs.
	17.253	0.95	465.043	78.80126	0.107	1.2	243234	3,574	
	17.581	0.97	6188.606	667.96301	1.430	0.9	56705	30,299	1.480 02
H-2	18.078	1.00	425316.972	39229.175 68	98.245	0.7	66541	1,779,4 21	1.707 93
	18.387	1.02	271.798	33.44834	0.063	1.4	70731	1,517	0.840 90
	18.651	1.03	673.216	92.02648	0.156	1.0	218212	4,174	0.840 13
		Sum	432915.6339						

Signal: MS1 +TIC SCAN ESI Frag=135V Gain=1.0

Name	RT [min]	RRT	Area	Height	Area%	TF	Plates	S/N	Rs.
H-2-	18.360	1.00	1095383858. 134	22948336.1 5574	100.000	0.7	3467	575	
		Sum	1095383858. 1335						

Figure S3. High-performance liquid chromatography (HPLC) analysis of synthesized C14-192 cationic lipid compound.

a

	LNP (molar ratio)	mRNA	Diameter (nm) ± SD	PDI	ZP (mV)	Encapsulation Efficiency
1	SM102 : DSPC : Cholesterol : DMG-PEG 2000 = 50 : 10 : 38.5 : 1.5	Luciferase	119.84 ± 39.03	0.102	3.33	93%
2	C14-192 : DSPC : Cholesterol : DMG-PEG 2000 = 50 : 10 : 38.5 : 1.5	Luciferase	130.44 ± 43.15	0.126	1.20	91%
3	C14-192 : DSPC : Cholesterol : DMG-PEG 2000 = 45 : 8 : 45.5 : 2	Luciferase	131.07 ± 53.47	0.118	1.37	90%
4	C14-192 : DSPC : β-Sitosterol : DMG-PEG 2000 = 45 : 8 : 45.5 : 2	Luciferase	133.06 ± 53.08	0.175	1.80	90%

b

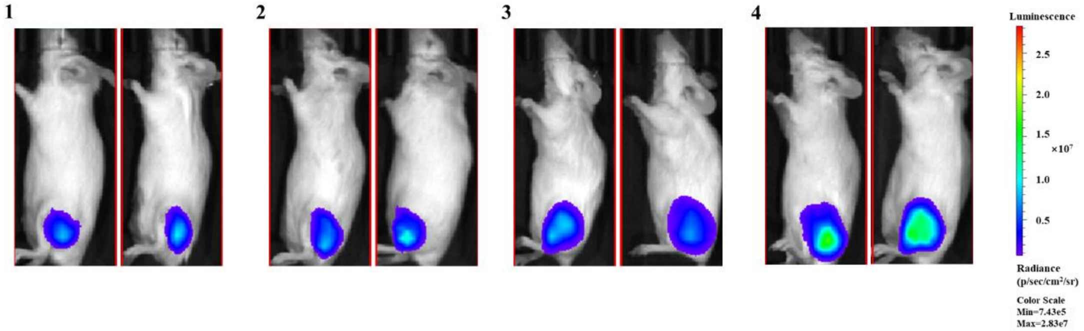

Figure S4. Characterization and analysis of the in vivo delivery efficiency of lipid nanoparticles (LNPs) with different components or molar ratios. (a) Characterization of four LNPs with varying lipid components or molar ratios, including size distribution, polydispersity index (PDI), zeta potential (ZP), and encapsulation efficiency. (b) Fluorescence intensity image of mice injected with the indicated luciferase mRNA-LNPs for 24 hours. Representative results are presented as means ± standard deviation (SD).

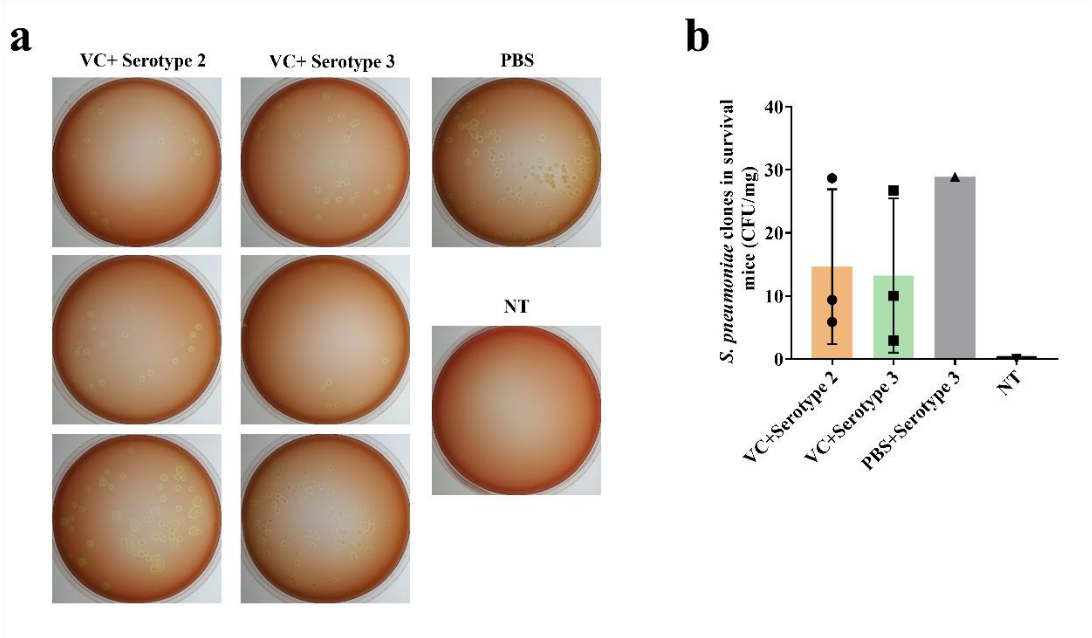

Figure S5. Bacterial load assay for lung tissue from representative survival mice pre-vaccinated with Vaccine C (VC) and infected with lethal dose of *S. pneumoniae* serotype 2 or serotype3, showing (a) the representative captures and (b) the average number of bacterial clones in Columbia blood agar plates. Representative results are shown as mean ± SD.
