# Supplementary material for: Development of a novel-ionizable-lipid-based mRNA vaccine for broad protection against Streptococcus pneumoniae
Source: Mol Ther Nucleic Acids. 2025 Sep 1;36(4):102699. doi: 10.1016/j.omtn.2025.102699 (PMC12744836; doi:10.1016/j.omtn.2025.102699)
Supplement: Document S2. Article plus supplemental information [file mmc2.pdf]

Molecular Therapy
Nucleic Acids
Original Article

Development of a novel-ionizable-lipid-based mRNA vaccine for broad protection against *Streptococcus pneumoniae*

Shi Xu,[1,5] Guoqing Qi,[2,3,5] Rui Liu,[1,5] Shang Liu,[1] Aili Wang,[1] Wei Li,[1] Keyue Ruan,[1] Lingzhi Zhan,[1] Lianshun Wang,[1] Caiyi Fei,[1] Jiyang Zhao,[4] Xue Zhang,[4] Qin Yu,[4] Mengwei Xu,[1] Jing Li,[1] and Tiyun Han[1]

[1]Nanjing Chengshi (TheraRNA) Biomedical Technology Co. Ltd., Nanjing 210000, Jiangsu, China; [2]The Second Clinical Medical College, Lanzhou University, Lanzhou 730030, Gansu, China; [3]Department of Gastroenterology, Lanzhou University Second Hospital, Lanzhou 730030, Gansu, China; [4]School of Environmental Science, Nanjing Xiaozhuang University, Nanjing 211171, Jiangsu, China

Recently, messenger RNA (mRNA)-based vaccine technology has made significant advances in preventing pathogenic microbial infections. The composition and physicochemical properties of lipid nanoparticle (LNP) determine the delivery efficiency of mRNA vaccines. In this study, we synthesized a novel ionizable lipid, C14-192, featuring a 3-oxo-polyamine head group, which was used as a component for LNP to encapsulate and deliver mRNA. Analysis of *in vitro* and *in vivo* expression showed that C14-192-LNP-encapsulated luciferase mRNA exhibited high expression efficiency. To further assess the potential of the C14-192 LNP formulation for vaccine applications, we developed a prophylactic mRNA vaccine against *Streptococcus pneumoniae* (*S. pneumoniae*), based on the conserved and truncated pneumococcal histidine triad protein D (PhtD) and pneumolysin (Ply). The mRNA encoding the fusion construct exhibited the highest expression and secretion levels. In murine model, mRNA vaccine effectively prevented *S. pneumoniae* infection and colonization in the lungs and prevented severe lesions. Moreover, the vaccine demonstrated robust cross-protection against multiple serotypes of *S. pneumoniae* and provide effective protection against lethal infection. In conclusion, a novel ionizable lipid was successfully synthesized and applied in the development of a new prophylactic vaccine against *S. pneumoniae*.

INTRODUCTION

Messenger RNA (mRNA) vaccines have achieved remarkable progress in both therapeutic and clinical researches, demonstrating potential for the prevention of viral and bacterial infections, cancer immunotherapy, or other diseases.[1–3] The emergence of lipid nanoparticle (LNP)-encapsulated mRNA has fundamentally transformed vaccine development by offering high immunogenicity, rapid manufacturing, and a well-characterized safety profile that is generally favorable compared to viral vectors and traditional adjuvant-based vaccines.[4] This improved safety profile refers specifically to the absence of insertional mutagenesis risk, better control of dose and expression, and a lower incidence of vector-related adverse events as documented in previous clinical trials.[5]

LNPs function as delivery vehicles for nucleic acids. They are typically composed of ionizable lipids, helper phospholipids, cholesterol, and polyethylene glycol (PEG), which together determine the particle's encapsulation efficiency, biodistribution, and immunostimulatory properties.[5,6] Among these, ionizable lipids and auxiliary phospholipids primarily form the nanoparticle core, achieving stable encapsulation and efficient endosomal escape.[6,7] After administration, LNPs are taken up by cells via endocytosis and release their mRNA cargo into the cytoplasm for antigen expression. Compared to viral vectors or polymer-based nanoparticles, LNPs offer advantages of lower genotoxicity risk, flexible manufacturing, and reproducible batch quality.[5]

However, conventional LNP systems can have limitations, including a tendency for hepatic accumulation, suboptimal delivery efficiency to target tissues, and unwanted inflammatory responses.[5,8] Previous studies have shown that changing the lipid composition or making specific structural modifications on lipid can markedly alter the physicochemical characteristics, organ distribution, and immunogenicity of LNPs.[9,10] Recent advances in the design of ionizable lipids—for example, using refined structures like (4S)-KEL—have demonstrated enhanced mRNA encapsulation, organ-selective delivery, and a more favorable balance between efficacy and inflammatory

Received 30 May 2025; accepted 28 August 2025;
https://doi.org/10.1016/j.omtn.2025.102699.

[5]These authors contributed equally

Correspondence: Mengwei Xu, Nanjing Chengshi (TheraRNA) Biomedical Technology Co. Ltd., Nanjing 210000, Jiangsu, China.
E-mail: xu.mengwei@therarna.cn
Correspondence: Jing Li, Nanjing Chengshi (TheraRNA) Biomedical Technology Co. Ltd., Nanjing 210000, Jiangsu, China.
E-mail: li.jing@therarna.cn
Correspondence: Tiyun Han, Nanjing Chengshi (TheraRNA) Biomedical Technology Co. Ltd., Nanjing 210000, Jiangsu, China.
E-mail: hantiyun@therarna.cn

© 2025 The Author(s). Published by Elsevier Inc. on behalf of The American Society of Gene and Cell Therapy. This is an open access article under the CC BY license (http://creativecommons.org/licenses/by/4.0/).

response compared to first-generation lipids such as SM-102.[11,12] This comparison emphasizes that improvements in delivery efficiency or safety must always be benchmarked against clinically validated LNPs, such as ALC-0315 and SM-102, which are widely used in authorized mRNA vaccines. Additionally, a new class of ionizable cationic lipids, 1- and 2-oxo-polyamine lipids, has been explored for LNP formulation.[13] These lipids can be synthesized without extra solvents or protecting group steps, simplifying manufacturing within a few days. Studies indicate that adding oxygen atoms to the polyamine head groups may enhance LNP chemical stability and reduce potential cytotoxicity.[14] Nonetheless, whether introducing a higher degree of oxygenation in the oxo-polyamine head can further improve biocompatibility and expression efficiency remains to be systematically studied.

In recent years, mRNA vaccines have attracted growing interest for bacterial infection prevention and treatment.[15] As with viral mRNA vaccines, the immunization efficacy depends not only on the selected antigen but also critically on the delivery efficiency determined by the LNP carrier.[16] The translation of mRNA-LNP vaccines against multidrug-resistant (MDR) pathogens faces specific challenges, including insufficient delivery performance, inflammatory reactogenicity associated with conventional LNP formulations, and limited antigen breadth.[17,18] These challenges underscore the need to optimize both the LNP composition and the choice of conserved antigens with strong cross-reactivity.

MDR bacterial infections, such as those caused by *Streptococcus pneumoniae* (*S. pneumoniae*), present significant global health concerns due to high antigenic diversity and rising antibiotic resistance. Conventional protein-based vaccines often fail to provide cross-protection across serotypes, necessitating the development of novel antigen selection strategies.[19] The existing pneumococcal vaccines, including the pneumococcal polysaccharide vaccine (PPV) and pneumococcal conjugate vaccine (PCV) vaccines, are only moderately effective against specific serotypes, and they can be costly and vulnerable to serotype replacement.[19,20] Conserved pneumococcal virulence factors, such as pneumococcal histidine triad protein D (PhtD) and pneumolysin (Ply), have emerged as antigen candidates due to their critical roles in bacterial pathogenesis and high sequence conservation across diverse strains.[21–23] Integrating these conserved proteins into a mRNA-LNP vaccine could elicit synergistic protective responses, combining neutralizing antibodies with Th1-biased cellular immunity to counter heterologous pneumococcal infections.[22,23] Evidence from multivalent mRNA-LNP vaccines for viral infections supports the feasibility of such approaches for bacterial targets as well.[18,24]

In this study, we developed a novel ionizable lipid containing a 3-oxo-polyamine head group with multiple hydroxyl groups, designated as C14-192, which was designed to enhance mRNA expression efficiency and reduce potential cytotoxicity. Using C14-192 LNP, we formulated an optimized LNP to deliver an mRNA encoding a fusion antigen composed of the conserved N-terminal region of PhtD and C-terminal region of Ply. The fusion mRNA construct demonstrated higher secretion levels *in vitro*. In mouse model of *S. pneumoniae* infection, the novel mRNA vaccine significantly reduced bacterial burden in the lungs, alleviated lung inflammation and tissue injury, and provided cross-protection against multiple serotypes, including lethal challenge. These findings highlight the potential of combining structure-optimized ionizable lipids with conserved antigen design to advance mRNA vaccine development against MDR bacterial pathogens.

RESULTS

Synthesis of a novel epoxide-derived cationic lipid and evaluation for delivery

Recent studies have focused on improving the delivery and expression efficiency of mRNA through the optimization of ionizable lipids in LNPs. Previously, a library of lipid-like compounds was synthesized via epoxide chemistry to identify materials capable of targeting and expressing *in vivo* for gene silencing.[13] Similarly, we successfully synthesized a novel cationic lipid, C14-192, through the ring-opening of epoxides with amine substrates (Figure 1A). This compound contains abundant hydroxyl groups, which may enhance its biocompatibility and delivery efficiency. The structure of C14-192 was further characterized using proton nuclear magnetic resonance (1H NMR) spectroscopy and liquid chromatography-mass spectrometry (LC-MS) (Figures S1 and S2), with the purity of the synthesized lipid determined to be approximately 98% through high-performance liquid chromatography (HPLC) analysis (Figure S3). To test the lipid's potential for mRNA delivery, luciferase mRNA was prepared for encapsulation into LNP using a microfluidic process involving four lipid components (Figures 1B and 1C). We then compared the *in vivo* delivery efficiency of four different LNP formulations, each with varying lipid components and molar ratios (Figure S4A). By systematically altering the ionizable lipid, molar ratio, and cholesterol content, we identified the C14-192 LNP formulation, comprising C14-192, 1,2-distearoyl-sn-glycero-3-phosphocholine (DSPC), β-sitosterol, and 1,2-dimyristoyl-rac-glycerol-3-methoxypolyethylene glycol 2000 (DMG-PEG 2000) in a molar ratio of 45:8:45.5:2, as the most effective for further vaccine development. This formulation exhibited the highest *in vivo* mRNA expression levels (Figure S4B).

We next performed physicochemical characterization of the selected C14-192 LNP and compared it to SM102 LNP, a widely used formulation. The diameter distribution and polydispersity index (PDI) values indicated that while the uniformity of the C14-192 LNP was somewhat lower than that of the SM102 LNP (Figures 1D and 1E), both formulations demonstrated encapsulation efficiencies of 90% or higher. The C14-192 LNP also exhibited a lower positive charge compared to the SM102 LNPs (Figure 1E). Transmission electron microscopy (TEM) imaging confirmed that the mRNA-LNP complexes were intact and uniform (Figure 1F). *In vitro* transfection assays revealed that C14-192-LNP-encapsulated luciferase mRNA resulted in higher expression levels than SM102 LNP (Figure 1G). This enhanced expression was further confirmed in *in vivo* delivery

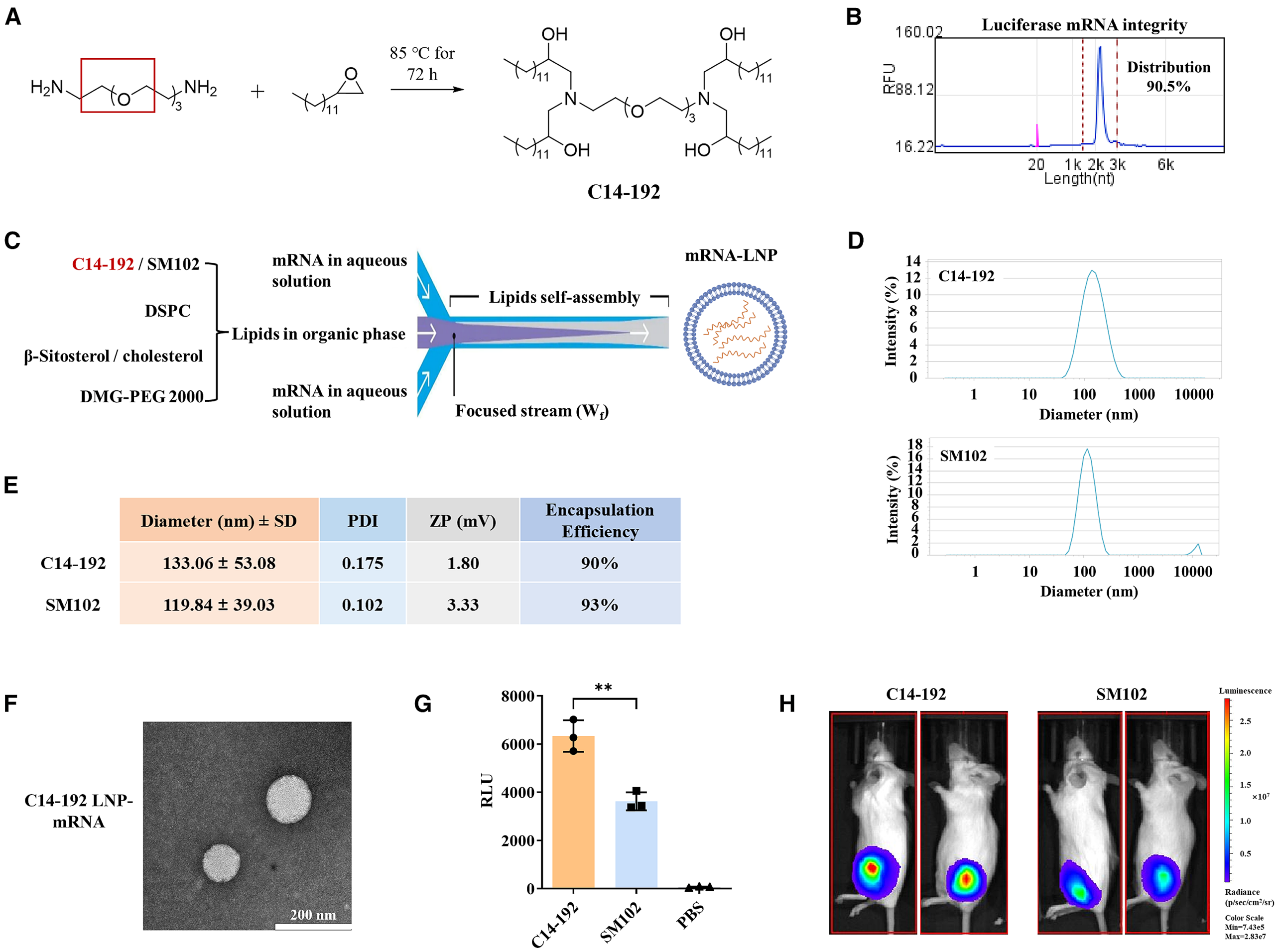

Figure 1. Synthesis of a novel ionizable lipid for LNP encapsulation of mRNA and antigen expression efficiency

(A) Schematic representation of the chemical synthesis of the novel ionizable lipid, C14-192. (B) Capillary electrophoresis analysis for the prepared luciferase mRNA. (C) Schematic of mRNA-LNP preparation using microfluidic device. (D) Particle size distribution of C14-192 and SM102 mRNA-LNPs. (E) Characterization of mRNA-LNPs containing C14-192 or SM102, including diameter, PDI, ZP, and encapsulation efficiency. (F) TEM image of the C14-192 LNP-mRNA complex. (G) Relative light unit (RLU) representing the fluorescence intensity after transfection of luciferase mRNA to HEK293T for 24 h. (H) Image of the fluorescence intensity for mice injected with different luciferase mRNA-LNP for 24 h. Representative results (n = 3) are presented as means ± standard deviation (SD). Scale bar, 200 μm. Values of $^{**}p < 0.01$ was considered significant.

experiments (Figure 1H). In conclusion, we successfully synthesized a novel ionizable lipid, C14-192, featuring a 3-oxo-polyamine head group via a one-step synthesis method. This lipid demonstrated optimal physicochemical properties and significantly enhanced mRNA expression efficiency, showing great potential for use in the development of mRNA vaccines.

Development of a novel mRNA vaccine against *S. pneumoniae* based on C14-192 LNP

To further evaluate the potential of C14-192 LNP for mRNA vaccine development, we used a mouse model of *S. pneumoniae* infection. Previous studies have shown that the Ply and PhtD proteins expressed by *S. pneumoniae* induce robust immune responses. Ply, as a virulence factor, triggers apoptosis and suppresses immune responses in host cells, while PhtD plays a role in pathogen adhesion and immune evasion.[25,26] Notably, both proteins exhibit high sequence conservation across various serotypes, highlighting their potential as broad-spectrum vaccine candidates. However, due to the toxicity of Ply and the large molecular size of PhtD, neither protein is suitable for full-length expression in a vaccine preparation.

In this study, we developed an mRNA vaccine by combining truncated versions of Ply and PhtD, incorporating conserved epitopes (Figure 2A). The C-terminal domain of Ply and the N-terminal domain of PhtD, along with a fusion protein of these two fragments, were selected for the mRNA constructs. As shown in Figure 2B, the truncated polypeptides maintained a complete three-dimensional structure after fusion, with minimal structural changes predicted in the model. The mRNA vaccines against *S. pneumoniae* were developed based on two truncated peptide sequences and fusion of them

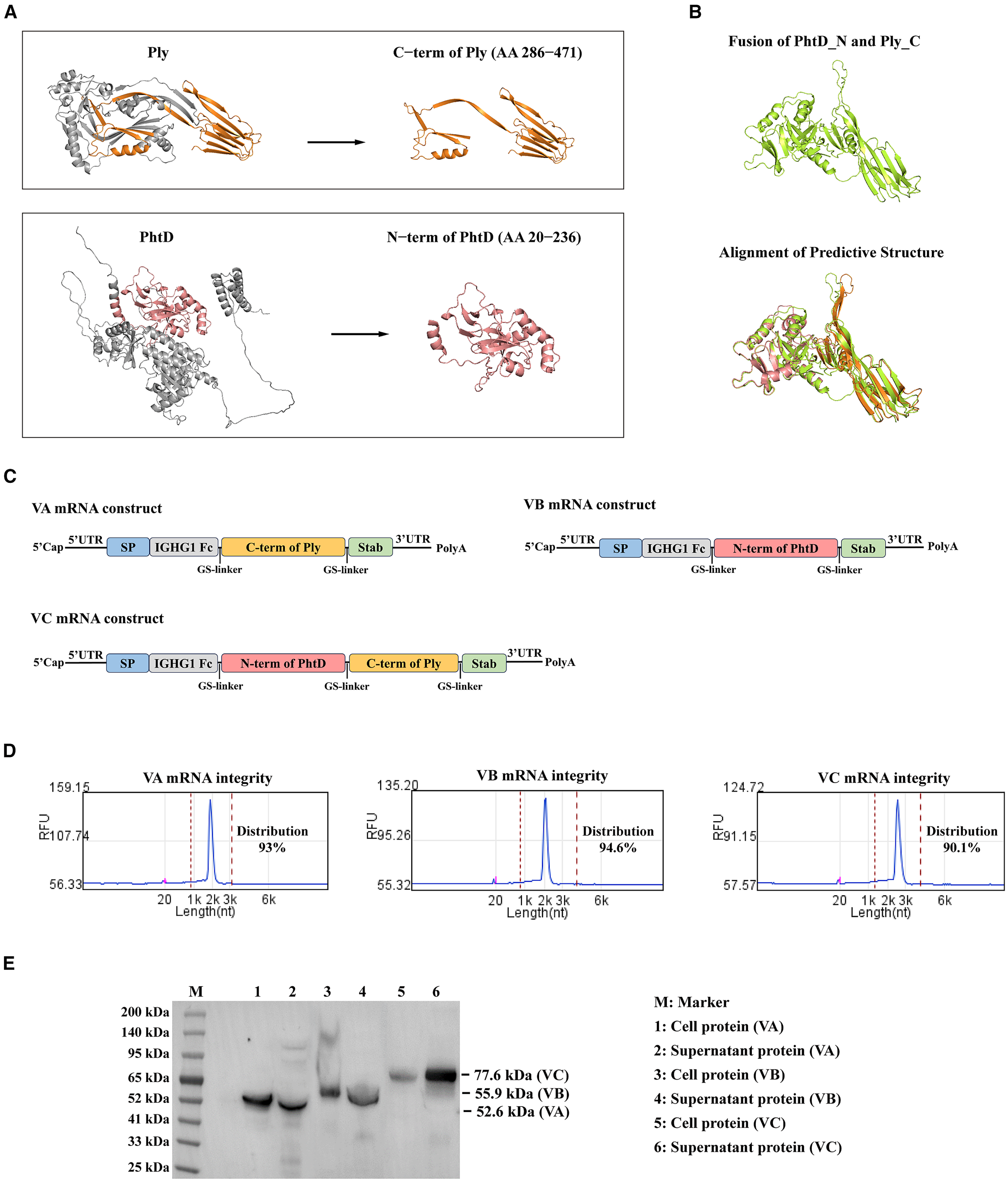

Figure 2. Development of mRNA vaccines against *S. pneumoniae* based on the conserved PhtD and Ply antigens

(A) Schematic representation of the secondary structure of Ply and PhtD, with the C-terminal of Ply and the N-terminal of PhtD highlighted in orange and pink, respectively. (B) Structure prediction of the PhtD-Ply fusion protein using AlphaFold3 and alignment with the corresponding truncated fragments. (C) Schematic representation of plasmid construction for vaccines VA, VB, and VC. (D) Capillary electrophoresis analysis for the indicated mRNA. (E) WB analysis of expressed antigens in cells and supernatants following transfection with the indicated mRNA.

(Figure 2C). The expression and secretion levels of the antigens were significantly influenced by the mRNA construct design. It has been shown that the Fc fragment of the human immunoglobulin gamma 1 heavy chain (IGHG1) and the STABILON (Stab) sequence can improve antigen trafficking and stability.[27,28] Therefore, these two elements were incorporated into the mRNA constructs. As a result, three different mRNA constructs were prepared: vaccine A (VA), vaccine B (VB), and vaccine C (VC) (Figure 2C).

After preparation, the integrity of the mRNA was analyzed, and the purity of all constructs exceeded 90% (Figure 2D). *In vitro* expression confirmed that all antigens were successfully expressed both in cells and the supernatant (Figure 2E). Notably, the secretion levels of the fused antigen in VC were significantly higher than the intracellular expression levels, when compared to VA and VB. Given that antigen secretion plays a crucial role in activating the humoral immune response against pathogen infection, VC was selected for further evaluation in animal experiments as a vaccine candidate against *S. pneumoniae*.

Prophylactic effect of novel mRNA vaccine against *S. pneumoniae*

We next evaluated the prophylactic effect of the novel mRNA vaccine in a mouse model intranasally infected with *S. pneumoniae* serotype 2. The experimental design for vaccination and bacterial infection is outlined in Figure 3A. Following prime-boost vaccinations, serum immunoglobulin G (IgG) antibody titers were measured. Mice immunized with the VC vaccine exhibited a high level of IgG against the PhtD antigen, whereas the IgG titer against the Ply antigen was comparatively lower (Figure 3B). Three days after *S. pneumoniae* infection, the bacterial load in the lungs of VC-vaccinated mice was significantly reduced when compared to the phosphate-buffered saline (PBS)-injected control group. Bacterial colony counts revealed a remarkable 450-fold reduction (Figures 3C and 3D). Additionally, Figure 3D shows that in 80% of the vaccinated and infected mice, the bacterial load in lung tissue was extremely low (<10 colony-forming unit [CFU]/mg). Histopathological analysis of lung tissue further confirmed the protective effect of the VC vaccine. In PBS-treated mice, substantial infiltration of inflammatory cells in the alveoli was observed, along with localized hemorrhagic foci, significant thickening of the alveolar walls, and marked alveolar shrinkage (Figure 3E). In contrast, these pathological changes were significantly reduced in VC-vaccinated mice, where inflammation and damage to the alveolar structure were minimal.

Finally, the levels of inflammatory cytokines, including tumor necrosis factor alpha (TNF-α) and interleukin-1β (IL-1β), were significantly lower in the serum and bronchoalveolar lavage fluid (BALF) of VC-vaccinated mice (Figure 3F). These findings were consistent with the observed lung pathology. Collectively, these results suggest that the novel mRNA vaccine based on C14-192 LNP effectively prevents *S. pneumoniae* infection in mice and alleviates lung tissue damage.

Cross-protective effect of novel mRNA vaccine against different serotypes of *S. pneumoniae*

To assess whether the novel mRNA vaccine, developed using the C14-192 LNP formulation and conserved antigens, provides cross-protection against *S. pneumoniae* of different serotypes, we conducted *in vivo* challenge experiments using serotypes 5, 3, and 19A (Figure 4A). Lung bacterial burden assays following infection showed that the VC vaccine significantly inhibited infection and bacterial colonization in the lungs for all three *S. pneumoniae* serotypes (Figures 4B and 4C). Likewise, lung pathology analysis indicated that the vaccine effectively mitigated tissue damage caused by acute bacterial infection, offering protection to the lung tissue from significant damage (Figure 4D).

Prophylactic effect of mRNA vaccine against lethal *S. pneumoniae* infection

To further evaluate the prophylactic efficacy of the vaccine under conditions of severe infection, we established a lethal *S. pneumoniae* infection model. The immunization protocol was modified to include three vaccinations spaced 2 weeks apart (Figure 5A). In the case of lethal infection with serotype 2 S. pneumoniae, 80% of the unvaccinated mice succumbed by the end of the experiment, whereas no vaccinated mice died (Figure 5B). Similarly, in the lethal serotype 3 *S. pneumoniae* infection model, all unvaccinated mice died by day 5, while only one vaccinated mouse succumbed. These findings demonstrate that the vaccine provides significant protection against lethal *S. pneumoniae* infections in two different serotypes.

Furthermore, we assessed the pulmonary bacterial burden and pathology in representative survivors from the vaccinated groups. By the end of the experiment, two-thirds of the immunized and infected mice exhibited significantly lower bacterial loads in their lungs compared to the unvaccinated mice (Figure S5). Although lung pathology remained elevated in all treated groups, the single surviving unvaccinated mouse showed the most severe inflammation and tissue damage (Figure 5C). These results from the lethal infection challenge demonstrate that the vaccine not only inhibits bacterial colonization but also reduces the extent of severe pulmonary lesions, ultimately improving survival outcomes in the infected mice.

DISCUSSION

The delivery efficiency and safety of mRNA vaccines are critically dependent on the choice of LNP, with ionizable lipids serving as the key components for mRNA encapsulation, endosomal escape, and cellular transfection.[7,8,29] Previous studies have shown that optimizing ionizable lipids can significantly influence the expression and immunogenicity of mRNA vaccines by adjusting factors such as charge, head group structure, and chemical composition.[9,30] Notably, the incorporation of oxygen atoms into ionizable lipids has been demonstrated to improve the stability of LNP and reduce their toxicity.[13] In this study, we introduced a novel ionizable lipid, C14-192, a 3-oxo-polyamine compound, which exhibited superior antigen expression both *in vitro* and *in vivo*. The composition of the lipid components, including ionizable lipids, cholesterol, and

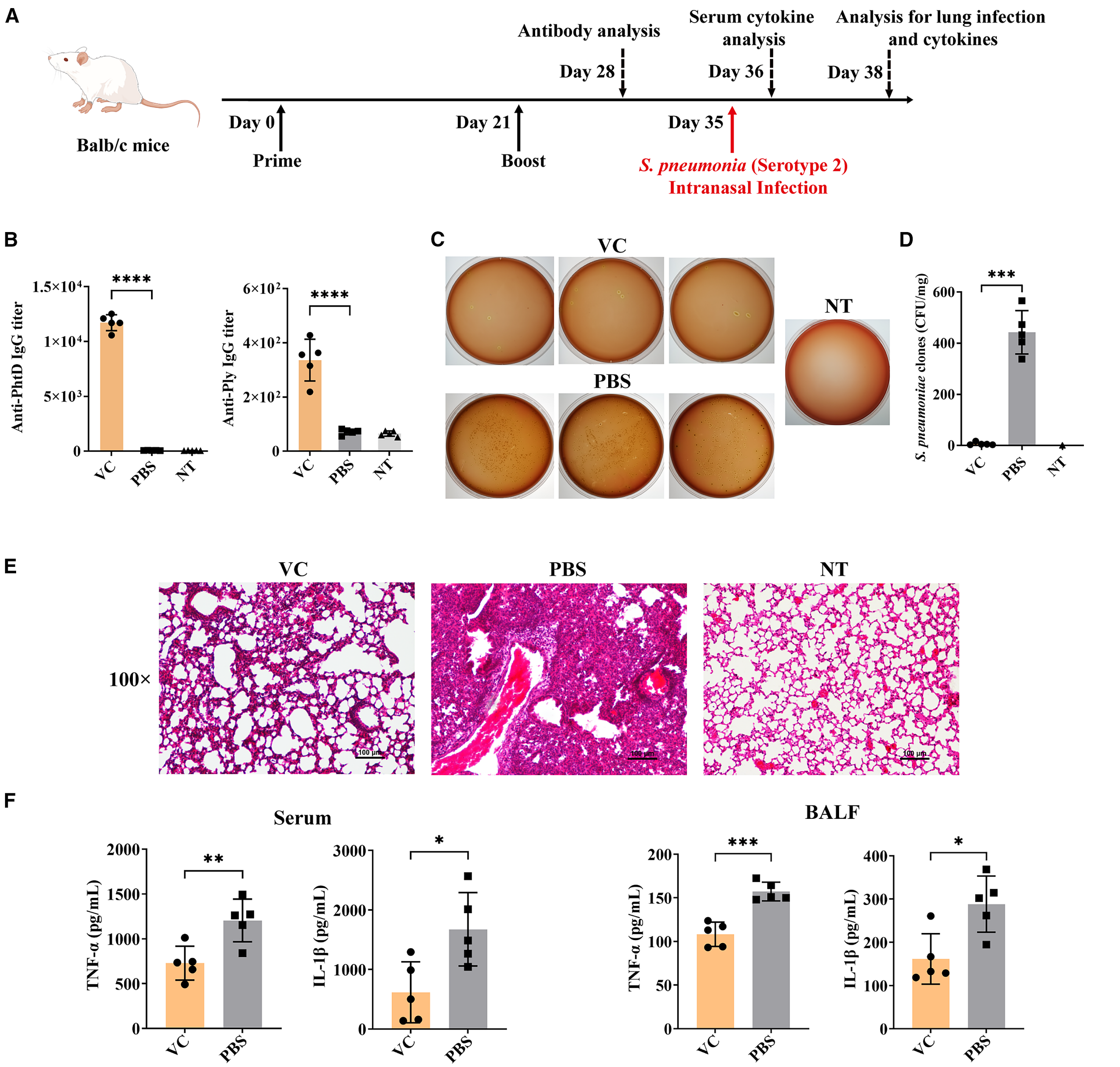

Figure 3. Prophylactic effect of PhtD-Ply mRNA vaccine in mice infected with *S. pneumoniae*

(A) Schematic of the experimental procedure for prophylactic mRNA vaccine and subsequent *S. pneumoniae* serotype 2 infection in mice. (B) Analysis of serum IgG antibody titers against PhtD or Ply antigen 28 days post-vaccination. (C and D) Bacterial load assay for lung tissue of mice on day 38, showing (C) the representative captures and (D) the average number of bacterial clones in Columbia blood agar plate. (E) Pathological analysis of lung tissues from experimental mice using H&E staining. (F) Cytokine analysis for serum samples on day 36 and bronchoalveolar lavage fluid (BALF) samples on day 38, including TNF-α and IL-1β. Representative results (n = 5) are shown as means ± SD. Values of *$p < 0.05$, **$p < 0.01$ and ***$p < 0.001$ were considered significant.

their molar ratios, can influence the physicochemical properties of LNP, which in turn affect their delivery efficiency. Interestingly, while the size distribution of C14-192 LNP was broader compared to SM102 LNP, this observation suggests that the uniformity and stability of C14-192 LNPs could be further improved. Enhancing these properties may ultimately lead to better delivery and expression outcomes.

Recent advancements in LNP formulation have focused on optimizing ionizable lipid structures to improve specificity, efficacy,

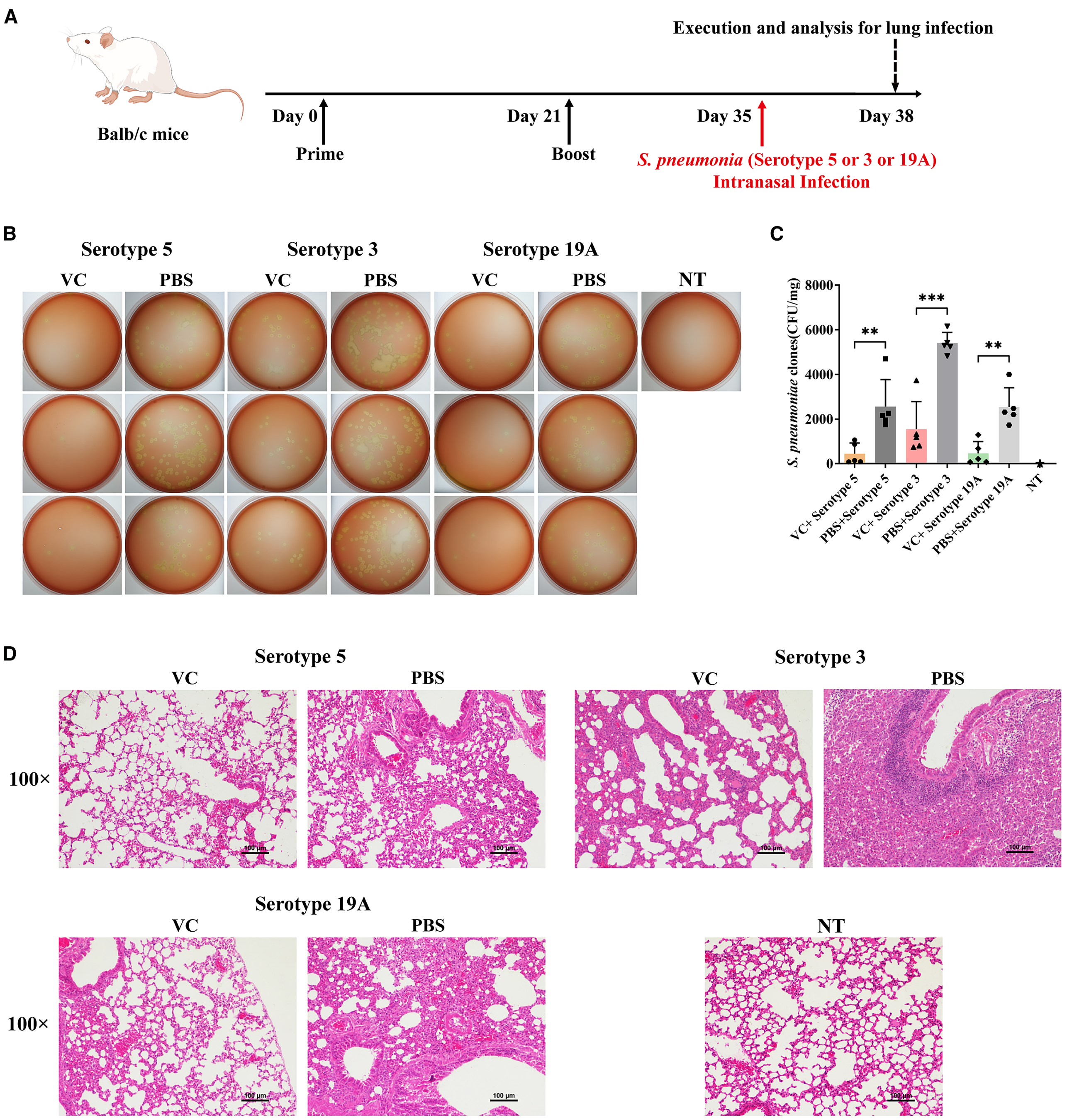

Figure 4. Prophylactic effect of PhtD-Ply mRNA vaccine against different serotypes of *S. pneumonia*

(A) Schematic of the experimental procedure for prophylactic mRNA vaccine and infection with different serotypes of *S. pneumoniae* in mice. (B and C) Bacterial load assay for lung tissue from mice on day 38, showing (B) the representative captures and (C) the average number of bacterial clones in Columbia blood agar plates. (D) Pathological analysis of lung tissues at the end of the experiment using H&E staining. Representative results ($n = 5$) are shown as means ± SD. Values of $^{**}p < 0.01$ and $^{***}p < 0.001$ were considered significant.

and biocompatibility.[11,31,32] Numerous studies have identified and tested novel ionizable lipids for applications in infectious diseases, cancer, and genetic disorders. For instance, CL15H6 LNPs, which were selected from a diverse library of ionizable lipids, demonstrated a high capacity for targeting splenic antigen-presenting cells.[33] These LNPs exhibited a significant reduction in tumor growth when used for delivering antigen-encoding mRNA. Additionally, A3B7C2 showed an 18.3-fold increase in mRNA transfection in dendritic cells

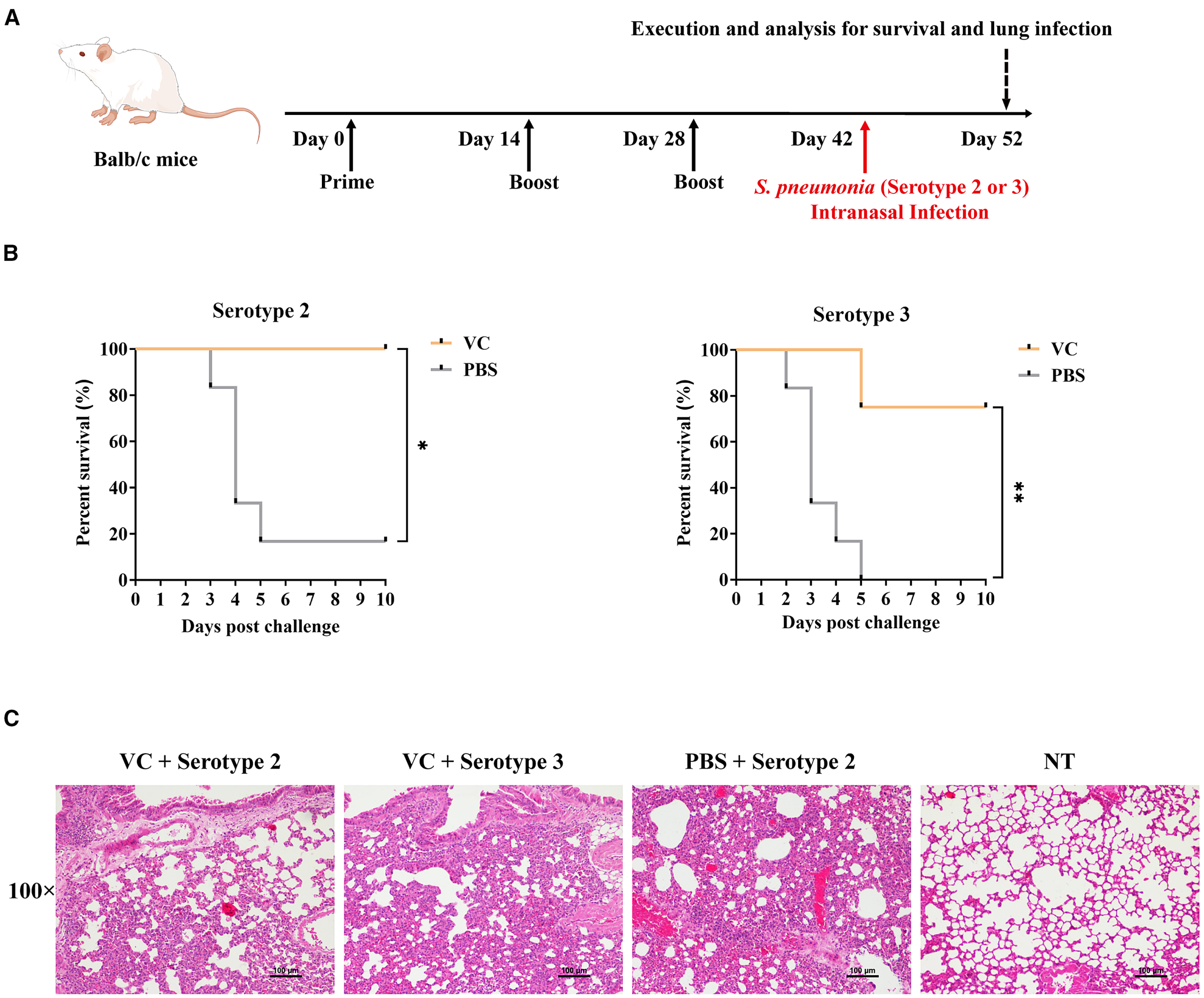

Figure 5. Prophylactic effect of PhtD-Ply mRNA vaccine against lethal *S. pneumoniae* infection
(A) Schematic of the experimental procedure for immunization with mRNA vaccine followed by infection with a lethal dose of *S. pneumoniae* serotype 2 or serotype 3 in mice. (B) Survival analysis of vaccinated mice on 10 days post-infection. (C) Pathological analysis of lung tissue from representative surviving mice at the end of the experiment using H&E staining. Values of $*p < 0.05$ and $**p < 0.01$ were considered significant.

compared to SM102, a critical feature for vaccines aimed at inducing robust humoral immunity.[34] For pulmonary delivery, IR-117-17 LNPs achieved a 300-fold higher mRNA expression in the lungs compared to conventional LNPs, highlighting its potential for inhaled vaccines targeting respiratory viruses.[35] In our study, C14-192 LNP, when used in the development of an mRNA vaccine against *S. pneumoniae*, demonstrated cross-protection against multiple serotypes in mice by encapsulating conserved antigens PhtD and Ply. This finding underscores the potential application of C14-192 and similar ionizable lipids in the development of mRNA vaccines for various pathogens. Notably, SM102 LNP was used as a control during characterization and expression experiments in this study. We compared delivery and expression efficiencies by altering the ionizable lipid, molar ratio, and cholesterol content. The final C14-192 LNP formulation, which exhibited the highest expression efficiency, was selected for the development of an mRNA vaccine to prevent bacterial infections.

MDR bacterial pathogens, such as *S. pneumoniae*, pose a significant clinical challenge due to their extensive serotype diversity and increasing antibiotic resistance, particularly among MDR strains.[36] Traditional polysaccharide-based vaccines, such as PPSV23 and PCV13, are limited by serotype-specific immunity, leaving populations vulnerable to non-vaccine serotypes.[36] This phenomenon of serotype replacement highlights the urgent need for next-generation vaccines capable of providing broad-spectrum protection.[37] mRNA-LNP vaccines offer a unique advantage in this regard, enabling the rapid development of vaccines targeting multiple conserved antigens

and enhancing antigen expression efficiency, thereby overcoming the limitations of serotype-specific immunity.[38] In this study, the dual-antigen mRNA vaccine targeting the conserved PhtD and Ply antigens exemplifies this approach. The vaccine significantly reduced bacterial colonization in the lungs and attenuated hyperinflammatory lung pathology, suggesting its potential for combating MDR pathogens.

An important consideration in the development of bacterial vaccines is the potential for post-translational modifications of the antigen expressed from the delivered mRNA in host cells. Unlike viral proteins, many bacterial proteins—such as PhtD and Ply—are not naturally glycosylated in their native bacterial context. However, when expressed in mammalian cells, the host cell may inadvertently add glycosylation modifications to recombinant bacterial proteins. These modifications can alter the structure of epitopes, reduce immunogenicity, or even mask critical antigenic sites.[39] Based on glycosylation prediction and amino acid sequence analysis, we found that the fusion construct does not contain canonical eukaryotic glycosylation motifs (N-X-S/T), which typically signal glycosylation sites. As a result, we did not introduce additional mutations to the construct. *In vitro* expression analysis confirmed the absence of glycosylation, which was evident from the appearance of two distinct protein bands. Additionally, previous studies have demonstrated that the Fc domain of IGHG1 and the STABILON sequence enhance antigen trafficking and stability. In our study, we incorporated these two elements directly into the mRNA constructs. Western blot (WB) analysis showed successful *in vitro* expression, particularly for the fusion antigen, which exhibited a higher secretion ratio compared to the single antigens. However, we observed multiple bands in the WB results, particularly for the N-terminal of PhtD in the supernatant. We speculate that this may be due to antibodies binding to degraded or aggregated protein molecules within the prepared samples.

Notably, although the PhtD-specific antibody titer was high following vaccination, the antibody response against Ply was relatively lower. We believe this difference in antibody responses may result from competition for immunodominant epitopes between the two antigens or from differences in antigen processing and presentation. Interestingly, the protective effect of the VC vaccine was not compromised by the relatively weaker antibody response to Ply. Antibodies against PhtD can directly block bacterial adhesion to or invasion of epithelial cells, playing a significant role in providing early-stage protection against infection. In contrast, the antibody response to Ply has a weaker direct impact on preventing bacterial colonization or adhesion during the early stages of infection. However, it may help mitigate the toxicity at later stages by neutralizing Ply's hemolytic activity against host cells.[40] Therefore, we conclude that the primary protective response of the fusion antigen is largely driven by PhtD, which functions mainly as a surface-adhesion-related protein exposed on the bacterial surface.

While this study represents a significant advancement, several limitations should be acknowledged. First, although C14-192 LNP demonstrates promising delivery efficiency, only one novel lipid was evaluated in the current study. Expanding the lipid library and conducting a comparative evaluation of multiple candidates under identical conditions would offer a more comprehensive understanding of performance variability. Second, while antigen expression and prophylactic efficacy were demonstrated, a more detailed analysis of the immune correlates of protection—such as antibody isotype profiles and T cell subset responses—is needed to better define the underlying mechanisms driving protection. Third, long-term safety and stability studies of the C14-192 LNP vaccine formulation in animal models, and eventually in non-human primates, are essential for advancing to clinical trials. Further optimization, in-depth immunological profiling, and clinical validation could establish C14-192-based mRNA vaccines as a powerful tool for combating other resistant bacterial infections. Finally, including an appropriate positive control, such as other pneumococcal vaccines or mRNA vaccines encapsulated by SM102 LNP, would be a reasonable next step.

In conclusion, this study developed a novel ionizable lipid as a highly effective component of LNP for mRNA delivery, which exhibited high antigen expression and provided protection against *S. pneumoniae*. The use of C14-192 combined with conserved antigens demonstrated high prophylactic efficacy across multiple *S. pneumoniae* serotypes. This work lays the foundation for broader applications of mRNA-LNP platforms in the fight against MDR infections and sets the stage for the development of next-generation vaccines.

MATERIALS AND METHODS

Ionizable lipid synthesis

In a 50 mL reaction vial, 10 mmol of 1,11-Diamino-3,6,9-trioxaundecane (BD196228, Bide Pharmatech, Shanghai, China), 50 mmol of 2-dodecyloxirane (BD53455, Bide Pharmatech), and 30 mL of dichloromethane were added sequentially. The reaction was then heated and stirred for 72 h at 85 °C. After the reaction was monitored by thin-layer chromatography (TLC), the sample was cooled down to room temperature (RT), and dichloromethane was removed by a rotary evaporator. The product was then separated on a silica gel column filled with silica (S5130, Sigma-Aldrich, USA) by TLC, with the eluent solution consisting of dichloromethane, methanol, and triethylamine in a volume ratio of 93:6:1, yielding a light-yellow, oily product, which is the novel cationic lipid component employed in this study, named 15,27-bis (2-hydroxytetradecyl)-18,21,24-trioxa-15,27-diazahentetracontane-13,29-diol (C14-192). Then, 1H NMR spectra were recorded on Bruker DRX500 (500 MHz) NMR spectrometers. Chemical shifts (δ) are reported in parts per million (ppm) relative to the resonance of tetramethylsilane (TMS) as the internal standard (0.00 ppm). Data are presented as follows: chemical shift, multiplicity (s = singlet, d = doublet, t = triplet, q = quartet, and m = multiplet), and integration. Then, the synthesized cationic liposome was further analyzed by LC-MS and HPLC (LC-ELSD-MS Agilent 1260&1290&6125C, Agilent Technologies, USA).

Structure prediction and alignment

The sequence information of PhtD (UniProt: Q8DQ08) and Ply (UniProt: Q7ZAK5) were acquired, and the structure information of PhtD (AlphaFold Protein Structure Database: AF-Q8DQ08-F1-v4) and Ply (AlphaFold Protein Structure Database: AF-Q7ZAK5-F1-v4) were acquired. The N-terminal (AA 20-236) of PhtD and C-terminal (AA 286-471) of Ply were marked with colors in PyMOL software (Schrödinger, NY, USA), respectively. The secondary structure of fused PhtD-Ply protein was predicted using AlphaFold 3 web tool (https://alphafoldserver.com). Finally, the alignment of predictive structure of PhtD-Ply with truncated fragment was made based on PyMOL software.

Plasmid construction

The pUC57 vector (GenScript) for mRNA *in vitro* transcription (IVT) was used for plasmid construction, and the T7 promoter element was introduced to enable efficient *in vitro* mRNA IVT. The vector also contained 5'-UTR, 3'-UTR and poly(A) tail of approximately 110 nucleotides to enhance mRNA stability and translation efficiency. Three constructs were designed, which included coding sequences (CDS) of C-terminal of Ply (VA), N-terminal of PhtD (VB), and fused PhtD-Ply protein with interval sequence (GGSGGGGSGG) in the middle (VC). The N-terminal also contains the signal peptide (SP) and the Fc domain of IGHG1, followed by GGS linker (GGSGGSGGSG) and antigen. The STABILON and 6×His-tag was co-expressed in C-terminal of antigen linked with 3×GS. The fragments were then cloned into the pUC57 vector, with a seamless cloning strategy being employed. The firefly luciferase CDS was cloned into a general pUC57 vector containing a T7 promoter, a 5'-UTR, a 3'-UTR, and a poly(A) tail.

mRNA synthesis

The template plasmid was digested to obtain a linearized plasmid for mRNA IVT, which was made with linearized plasmids using the MEGAscript T7 Transcription Kit through a modified protocol. Concurrently, mRNA was capped at the 5' terminus using the trinucleotide cap1 analog CleanCap (N-7413, TriLink). Following an incubation for 6 h and DNA template degradation, the IVT products were purified through lithium chloride. The mRNA concentration and purity were detected via NanoDrop (Thermo Fisher Scientific, Waltham, MA, USA). The average overall yield of mRNA IVT was 6–8 μg/μL in final products. The integrity of mRNA was detected using capillary electrophoresis, and the capping and tailing efficiencies were detected via LC-MS. The cap and tailing efficiencies averagely were 89%–95% and 85%–90%, respectively. The final product was stored at 4°C for rapid use.

mRNA-LNP preparation

The mRNA was encapsulated with LNP through microfluidics. For SM102 formulation, SM-102, DSPC, cholesterol, and DMG-PEG 2000 were dissolved in ethanol at a molar ratio of 50:10:38.5:1.5. In addition, C14-192, DSPC, cholesterol, and DMG-PEG2000 were dissolved in ethanol at a with molar ratio of 50:10:38.5:1.5 or 45:8:45.5:2, which was used to compare different C14-192 LNP formulations. For final cationic lipid formulation, C14-192, DSPC, β-sitosterol, and DMG-PEG2000 were dissolved in ethanol at a molar ratio of 45:8:45.5:2. In the meantime, the mRNA samples formulated into an aqueous phase solution at the desired final concentration in 20 mM sodium acetate buffer (pH 5.5). In microfluidic device, aqueous phase and organic phase solutions were mixed in the microfluidic chip, and the flow rate ratio of the aqueous phase to the organic phase was 95:5, and the weight ratio of the total lipids to the mRNAs was about 25:1, and the self-assembly of the positively charged lipids combined with negatively charged mRNAs was carried out. The obtained mRNA-LNP solution was adjusted to pH 7.0–7.4 with 1M Tris-HCI buffer (pH8.0), filtered through 0.22 μm filter membrane, and stored at 2 °C–8 °C. The particle size, PDI, and zeta potential (ZP) of LNP were measured using dynamic light scattering (DLS). The encapsulation efficiency was determined using the Quant-iT RiboGreen RNA Quantification Kit (R11490, Thermo Fisher Scientific) according to protocol. The mRNA-C14-192 LNP complex were observed via TEM in Zhenjiang Zhuanbo Testing Technology Co., Ltd.

In vitro expression of mRNA

For expression of firefly luciferase mRNA, HEK293T cells were seeded into 96-well plates at a density of 5×10^4 cells per well using MEM Basal Media (11095080, Gibco, MA, USA) containing 10% fetal bovine serum (FBS). After overnight incubation, mRNA-LNP (containing 0.33 μg mRNA) was added to the cells using Opti-MEM (31985070, Gibco). After a 24-h incubation, D-Luciferin, Sodium Salt (40901ES01, Yeasen, Shanghai, China) was introduced to the cells in accordance with protocol. The fluorescence intensity was quantified using a FlexStation 3 Multi-function Enzyme Labeler (Molecular Devices). For expression of VA, VB, and VC mRNA, HEK293T cells were seeded into T75 flask at a density of 4×10^6 cells using MEM Basal Media containing 10% FBS. Then, cells were transiently transfected with naked mRNA formulated in LNP3.[41] After 48 h of transfection, supernatants and cells were collected separately, and the latter were lysed using RIPA to obtain whole-cell proteins, and WB was performed to detect the expression levels of antigens.

Western blotting

Loading buffer (5×) was added to the cell lysates or the extracts and then incubated at 100 °C for 5 min. After that, it was centrifuged at 12,000 rpm for 5 min at 4°C. Then, 12% SmartPAG Precast Protein Gel Plus (SLE014, Smart-Lifesciences Biotech, Changzhou, China) was used, followed by the transfer of proteins to a PVDF membrane. The PVDF membrane was blocked with 5% skimmed milk powder at RT for 1 h and then incubated overnight at 4°C with an His-tag rabbit polyclonal antibody (LF308, Epizyme Biotech, Shanghai, China) diluted with General Antibody Diluent (1:1,000) (PS119L, Epizyme Biotech). After being washed three times with Tris-buffered saline with Tween 20 (TBST), the membrane was incubated with horseradish peroxidase (HRP)-conjugated goat anti-rabbit IgG (H+L) (LF102, Epizyme Biotech), diluted in General Antibody Diluent (1:5,000) at RT for 1 h. Then it was washed three times with TBST and analyzed using Omni-ECL Enhanced Detection Reagent

(SQ101L, Epizyme Biotech) in a fully automated chemiluminescence image analysis system (Shanghai Tanon Science & Technology).

Animals

All mice were purchased from Charles River Laboratories, Beijing, China. The experimental protocols for animals conformed to the Guidelines for the Care and Use of Laboratory Animals published by the National Institutes of Health. The animals were raised in a room where the air was kept moist (50%–60% humidity), and the light was on for 12 h and off for 12 h. They had free access to food and water. The animal experiments were conducted according to the national guidelines for the care and use of laboratory animals.

In vivo expression of luciferase mRNA-LNP

Six- to eight-week-old female Balb/c mice (18–25 g) were procured and acclimated to the facility environment. Firefly luciferase mRNA-LNP was injected into the mice at a dose of 5 μg of mRNA per mouse via the leg intramuscular injection, and the fluorescein substrate was injected into the peritoneal cavity of the mice 24 h later. Subsequent to 10 min, the fluorescence intensity at the injection site was captured with an animal imaging device (Lumina III, PerkinElmer, MA, USA).

Vaccination and *S. pneumoniae* infection

Six- to eight-week-old female Balb/c mice (18–25 g) were procured and randomly divided into different groups ($n = 5$) after acclimating to the facility environment. For the vaccination and *S. pneumoniae* serotype 2 (strain D39/NCTC7466) infection experiment, each mouse received a subcutaneous injection of either 5 μg of VC or 100 μL of PBS in the left groin on days 0 and 21. Then, on day 28, serum samples were collected for antibody titer analysis. On day 35, the mice were anesthetized with isoflurane and received a 40-μL solution containing 2×10^5 CFU of *S. pneumoniae* intranasally. Mice were then allowed to reside in a standard environment and were examined regularly for body weight, appearance, and mental status. On day 36, serum samples were collected for cytokine analysis. On day 38, BALF was collected for cytokine analysis. Part of the lung tissue was used for flow cytometry analysis of immune cell infiltration. Additionally, some lung tissue was used for bacterial load analysis and some was fixed in 4% paraformaldehyde for hematoxylin and eosin (H&E) staining and pathological analysis. Non-treated (NT) mice that were neither vaccinated nor infected were used as the blank control.

In the cross-protection study of the VC, multiple serotypes of *S. pneumoniae*, including serotypes 5 (strain BNCC367810), 3 (strain BNCC360205), and 19A (strain BNCC360196), were used to infect mice on day 35. The mice were vaccinated with mRNA vaccines or PBS on days 0 and 21. On day 38, the mice were euthanized, and their lung tissues were collected for bacterial load and pathological analyses.

To determine the lethal dose of *S. pneumoniae* infection, the mice were vaccinated on days 0, 14, and 28. Then, 40 μL of *S. pneumoniae* bacterial solution containing serotype 2 (6×10^8 CFU) or serotype 3 (2×10^8 CFU) was dropped intranasally. The mice were observed for 10 days. Percent survival analysis was performed, and pathological analysis of lung tissue from representative surviving mice was conducted at the end of the experiment.

Bacterial load assay for lung tissue

On day 38, the lung tissue from each group of mice was collected, cut into 10 mg pieces, and placed in sterile conditions. Then, 100 μL of sterile PBS was added. The tissue was then ground on ice until no obvious precipitation remained. Next, it was diluted 10-fold with sterile PBS. After thoroughly mixing, 100 μL of the dilution was spread evenly on a Columbia blood agar plate. The plate was then placed in a 37°C incubator for 48 h. After that, the bacterial clones on the plate were counted and photographed.

Indirect enzyme-linked immunosorbent assay for antibody titers

Serum PhtD-antigen-specific IgG antibody titers were determined by indirect enzyme-linked immunosorbent assay (ELISA). The steps are as follows: the microplate was coated with PhtD antigen or Ply antigen expressed and purified from *Escherichia coli* and incubated overnight at 4 °C; after washing three times with PBST, 3% BSA was added to each well and blocked at RT for 2 h; after washing three times, 100 μL/well of serially diluted serum was added to each well and incubated at RT for 2 h with gentle shaking; after washing three times, 100 μL/well of HRP-labeled rabbit anti-mouse IgG at a dilution of 1:5,000 was added and incubated at RT for 2 h with gentle shaking; the wells were washed three times and 100 μL/well of 3,3',5,5'-Tetramethylbenzidine (TMB) One Solution (36602ES60, Yeasen) was added and allowed to react at RT for 15 min in the dark; finally, 50 μL/well of 2 M H_2SO_4 solution was added to stop the reaction; the optical density (OD) value was measured at 450 nm using an enzyme marker (Multiskan FC, Thermo Fisher Scientific).

Enzyme-linked immunosorbent assay for cytokines

Serum samples were diluted 10-fold, and BALF was left untreated, followed by ELISA. The assay was performed according to the instructions for the Mouse TNF-α ELISA Kit (EA100124, OriGene, MD, USA) and the Mouse IL-1β ELISA Kit (EA100134, OriGene). Prepare the gradient dilution standards, add 100 μL each of standards and samples to the enzyme plate, and incubate at 37 °C for 90 min. Discard the samples, add 100 μL of biotinylated antibody working solution to each well, and incubate at 37°C for 60 min. Then, 100 μL of enzyme conjugate working solution was added to each well and incubated at 37 °C for 30 min. The reaction was terminated by adding 90 μL of TMB substrate solution to each well and incubated at 37 °C for 15 min, protected from light, and terminated by adding 50 μL of termination solution to each well. Finally, the OD value of each well was measured at 450 nm using an enzyme marker.

Statistical analysis

The differences between two groups were compared via the unpaired Student's t test. The differences among multiple groups were

analyzed by one-way analysis of variance (ANOVA) with Dunnett's test or, if appropriate, repeated-measures ANOVA with Bonferroni's post hoc correction. Values of $^{*}p < 0.05$, $^{**}p < 0.01$, $^{***}p < 0.001$, and $^{****}p < 0.0001$ were considered significant.

DATA AVAILABILITY

The datasets are available from the corresponding author upon reasonable request. The GenBank IDs of the constructed gene expression sequences are listed below: Ply_Cter-mRNA_construct (GenBank: PV692084), PhtD_Nter-mRNA_construct (GenBank: PV692085), and PhtD_Nter-Ply_Cter-mRNA_construct (GenBank: PV692086).

ACKNOWLEDGMENTS

This research was funded by The Natural Science Foundation of Gansu Province (No. 25JRRA611). This study was also supported by Nanjing Chengshi (TheraRNA) Biomedical Technology Co. Ltd.

AUTHOR CONTRIBUTIONS

M.X., J.L., S.X., and T.H. conceived and supervised the project. S.X., G.Q., and R.L. designed and performed the majority of experiments, analyzed data, and drafted the manuscript. R.L., W.L., and S.L. assisted with the mRNA vaccine construction and LNP formulation. L.W., L.Z., J.Z., and M.X. assisted with the animal immunization experiments, challenge studies, and pathological analysis. A.W., K.R., L.Z., X.Z., and Q.Y. performed the antibody assay. W.L., A.W., C.F., J.Z., X.Z., and Q.Y. contributed to data analysis and interpretation. M.X., J.L., G.Q., and T.H. reviewed and edited the manuscript. All authors reviewed and approved the final version of the manuscript.

DECLARATION OF INTERESTS

S.X., R.L., S.L., A.W., W.L., K.R., L.Z., L.W., C.F., M.X., J.L., and T.H. are full-time employees of Nanjing Chengshi (TheraRNA) Biomedical Technology Co. Ltd. Nanjing Chengshi (TheraRNA) Biomedical Technology Co. Ltd. has filed patent for pneumococcal mRNA vaccine, listing T.H., S.X., J.L., and M.X. as co-inventors. Nanjing Chengshi (TheraRNA) Biomedical Technology Co. Ltd. has filed patent for C14-192 LNP, listing R.L. and W.L. as co-inventors. The study was partially funded by Nanjing Chengshi (TheraRNA) Biomedical Technology Co. Ltd., which provided experimental, instrumental, and material support.

SUPPLEMENTAL INFORMATION

Supplemental information can be found online at https://doi.org/10.1016/j.omtn.2025.102699.

REFERENCES

1. Sun, X., Setrerrahmane, S., Li, C., Hu, J., and Xu, H. (2024). Nucleic acid drugs: recent progress and future perspectives. Signal Transduct. Tar. *9*, 316. https://doi.org/10.1038/s41392-024-02035-4.
2. Chaudhary, N., Weissman, D., and Whitehead, K.A. (2021). mRNA vaccines for infectious diseases: principles, delivery and clinical translation. Nat. Rev. Drug Discov. *20*, 817–838. https://doi.org/10.1038/s41573-021-00283-5.
3. Liu, C., Shi, Q., Huang, X., Koo, S., Kong, N., and Tao, W. (2023). mRNA-based cancer therapeutics. Nat. Rev. Cancer *23*, 526–543. https://doi.org/10.1038/s41568-023-00586-2.
4. Jackson, N.A.C., Kester, K.E., Casimiro, D., Gurunathan, S., and DeRosa, F. (2020). The promise of mRNA vaccines: a biotech and industrial perspective. NPJ Vaccines *5*, 11. https://doi.org/10.1038/s41541-020-0159-8.
5. Li, W., Wang, C., Zhang, Y., and Lu, Y. (2024). Lipid Nanocarrier-Based mRNA Therapy: Challenges and Promise for Clinical Transformation. Small *20*, e2310531. https://doi.org/10.1002/smll.202310531.
6. Li, B., and Dong, Y. (2017). Preparation and Optimization of Lipid-Like Nanoparticles for mRNA Delivery. Methods Mol. Biol. *1632*, 207–217. https://doi.org/10.1007/978-1-4939-7138-1_13.
7. Ma, Y., Chen, Y., Li, Z., and Zhao, Y. (2024). Rational Design of Lipid-Based Vectors for Advanced Therapeutic Vaccines. Vaccines (Basel) *12*, 603. https://doi.org/10.3390/vaccines12060603.
8. Escalona-Rayo, O., Zeng, Y., Knol, R.A., Kock, T.J.F., Aschmann, D., Slütter, B., and Kros, A. (2023). In vitro and in vivo evaluation of clinically-approved ionizable cationic lipids shows divergent results between mRNA transfection and vaccine efficacy. Biomed. Pharmacother. *165*, 115065. https://doi.org/10.1016/j.biopha.2023.115065.
9. Catenacci, L., Rossi, R., Sechi, F., Buonocore, D., Sorrenti, M., Perteghella, S., Peviani, M., and Bonferoni, M.C. (2024). Effect of Lipid Nanoparticle Physico-Chemical Properties and Composition on Their Interaction with the Immune System. Pharmaceutics *16*, 1521. https://doi.org/10.3390/pharmaceutics16121521.
10. Wang, C., Zhang, Y., and Dong, Y. (2021). Lipid Nanoparticle-mRNA Formulations for Therapeutic Applications. Acc. Chem. Res. *54*, 4283–4293. https://doi.org/10.1021/acs.accounts.1c00550.
11. Somu Naidu, G., Rampado, R., Sharma, P., Ezra, A., Kundoor, G.R., Breier, D., and Peer, D. (2025). Ionizable Lipids with Optimized Linkers Enable Lung-Specific, Lipid Nanoparticle-Mediated mRNA Delivery for Treatment of Metastatic Lung Tumors. ACS Nano *19*, 6571–6587. https://doi.org/10.1021/acsnano.4c18636.
12. Lv, K., Yu, Z., Wang, J., Li, N., Wang, A., Xue, T., Wang, Q., Shi, Y., Han, L., Qin, W., et al. (2024). Discovery of Ketal-Ester Ionizable Lipid Nanoparticle with Reduced Hepatotoxicity, Enhanced Spleen Tropism for mRNA Vaccine Delivery. Adv. Sci. *11*, e2404684. https://doi.org/10.1002/advs.202404684.
13. Love, K.T., Mahon, K.P., Levins, C.G., Whitehead, K.A., Querbes, W., Dorkin, J.R., Qin, J., Cantley, W., Qin, L.L., Racie, T., et al. (2010). Lipid-like materials for low-dose, in vivo gene silencing. Proc. Natl. Acad. Sci. USA *107*, 1864–1869. https://doi.org/10.1073/pnas.0910603106.
14. Zwolsman, R., Darwish, Y.B., Kluza, E., and van der Meel, R. (2025). Engineering Lipid Nanoparticles for mRNA Immunotherapy. Wiley Interdiscip. Rev. Nanomed. Nanobiotechnol. *17*, e70007. https://doi.org/10.1002/wnan.70007.
15. Imani, S., Lv, S., Qian, H., Cui, Y., Li, X., Babaeizad, A., and Wang, Q. (2025). Current innovations in mRNA vaccines for targeting multidrug-resistant ESKAPE pathogens. Biotechnol. Adv. *79*, 108492. https://doi.org/10.1016/j.biotechadv.2024.108492.
16. Tenchov, R., Bird, R., Curtze, A.E., and Zhou, Q. (2021). Lipid Nanoparticles horizontal line From Liposomes to mRNA Vaccine Delivery, a Landscape of Research Diversity and Advancement. ACS Nano *15*, 16982–17015. https://doi.org/10.1021/acsnano.1c04996.
17. Khlebnikova, A., Kirshina, A., Zakharova, N., Ivanov, R., and Reshetnikov, V. (2024). Current Progress in the Development of mRNA Vaccines Against Bacterial Infections. Int. J. Mol. Sci. *25*, 13139. https://doi.org/10.3390/ijms252313139.
18. Alameh, M.G., Semon, A., Bayard, N.U., Pan, Y.G., Dwivedi, G., Knox, J., Glover, R. C., Rangel, P.C., Tanes, C., Bittinger, K., et al. (2024). A multivalent mRNA-LNP vaccine protects against Clostridioides difficile infection. Science *386*, 69–75. https://doi.org/10.1126/science.adn4955.
19. Li, S., Liang, H., Zhao, S.H., Yang, X.Y., and Guo, Z. (2023). Recent progress in pneumococcal protein vaccines. Front. Immunol. *14*, 1278346. https://doi.org/10.3389/fimmu.2023.1278346.
20. Moffitt, K., and Malley, R. (2016). Rationale and prospects for novel pneumococcal vaccines. Hum. Vaccines Immunother. *12*, 383–392. https://doi.org/10.1080/21645515.2015.1087625.
21. Seiberling, M., Bologa, M., Brookes, R., Ochs, M., Go, K., Neveu, D., Kamtchoua, T., Lashley, P., Yuan, T., and Gurunathan, S. (2012). Safety and immunogenicity of a pneumococcal histidine triad protein D vaccine candidate in adults. Vaccine *30*, 7455–7460. https://doi.org/10.1016/j.vaccine.2012.10.080.
22. Verhoeven, D., Xu, Q., and Pichichero, M.E. (2014). Vaccination with a Streptococcus pneumoniae trivalent recombinant PcpA, PhtD and PlyD1 protein vaccine candidate protects against lethal pneumonia in an infant murine model. Vaccine *32*, 3205–3210. https://doi.org/10.1016/j.vaccine.2014.04.004.
23. Chan, W.Y., Entwisle, C., Ercoli, G., Ramos-Sevillano, E., McIlgorm, A., Cecchini, P., Bailey, C., Lam, O., Whiting, G., Green, N., et al. (2019). A Novel, Multiple-Antigen Pneumococcal Vaccine Protects against Lethal Streptococcus pneumoniae Challenge. Infect. Immun. *87*, e00846-18. https://doi.org/10.1128/IAI.00846-18.

24. Scott, N.R., Mann, B., Tuomanen, E.I., and Orihuela, C.J. (2021). Multi-Valent Protein Hybrid Pneumococcal Vaccines: A Strategy for the Next Generation of Vaccines. Vaccines (Basel) *9*, 209. https://doi.org/10.3390/vaccines9030209.

25. Oloo, E.O., Yethon, J.A., Ochs, M.M., Carpick, B., and Oomen, R. (2011). Structure-guided antigen engineering yields pneumolysin mutants suitable for vaccination against pneumococcal disease. J. Biol. Chem. *286*, 12133–12140. https://doi.org/10.1074/jbc.M110.191148.

26. Rioux, S., Neyt, C., Di Paolo, E., Turpin, L., Charland, N., Labbé, S., Mortier, M.C., Mitchell, T.J., Feron, C., Martin, D., and Poolman, J.T. (2011). Transcriptional regulation, occurrence and putative role of the Pht family of Streptococcus pneumoniae. Microbiology (Read.) *157*, 336–348. https://doi.org/10.1099/mic.0.042184-0.

27. Bournazos, S., and Ravetch, J.V. (2017). Fcgamma Receptor Function and the Design of Vaccination Strategies. Immunity *47*, 224–233. https://doi.org/10.1016/j.immuni.2017.07.009.

28. Rethi-Nagy, Z., Abraham, E., Udvardy, K., Klement, E., Darula, Z., Pal, M., Katona, R.L., Tubak, V., Pali, T., Kota, Z., et al. (2022). STABILON, a Novel Sequence Motif That Enhances the Expression and Accumulation of Intracellular and Secreted Proteins. Int. J. Mol. Sci. *23*, 8168. https://doi.org/10.3390/ijms23158168.

29. Gueguen, C., Ben Chimol, T., Briand, M., Renaud, K., Seiler, M., Ziesel, M., Erbacher, P., and Hellal, M. (2024). Evaluating how cationic lipid affects mRNA-LNP physical properties and biodistribution. Eur. J. Pharm. Biopharm. *195*, 114077. https://doi.org/10.1016/j.ejpb.2023.08.002.

30. Zhou, Y., Shan, X., Gu, R., Xia, Y., and Huang, X. (2025). Exploring the role of cationic lipids in modulating immunogenicity and vaccine efficacy of mRNA-LNP. Particuology *97*, 1–11. https://doi.org/10.1016/j.partic.2024.11.011.

31. Zhu, Y., Ma, J., Shen, R., Lin, J., Li, S., Lu, X., Stelzel, J.L., Kong, J., Cheng, L., Vuong, I., et al. (2024). Screening for lipid nanoparticles that modulate the immune activity of helper T cells towards enhanced antitumour activity. Nat. Biomed. Eng. *8*, 544–560. https://doi.org/10.1038/s41551-023-01131-0.

32. Wang, W., Chen, K., Jiang, T., Wu, Y., Wu, Z., Ying, H., Yu, H., Lu, J., Lin, J., and Ouyang, D. (2024). Artificial intelligence-driven rational design of ionizable lipids for mRNA delivery. Nat. Commun. *15*, 10804. https://doi.org/10.1038/s41467-024-55072-6.

33. Younis, M.A., Sato, Y., Elewa, Y.H.A., and Harashima, H. (2025). Harnessing the composition of lipid nanoparticles to selectively deliver mRNA to splenic immune cells for anticancer vaccination. Drug Deliv. Transl. Res. *15*, 3626–3641. https://doi.org/10.1007/s13346-025-01824-w.

34. Dong, W., Li, Z., Hou, T., Shen, Y., Guo, Z., Su, Y.T., Chen, Z., Pan, H., Jiang, W., and Wang, Y. (2024). Multicomponent Synthesis of Imidazole-Based Ionizable Lipids for Highly Efficient and Spleen-Selective Messenger RNA Delivery. J. Am. Chem. Soc. *146*, 15085–15095. https://doi.org/10.1021/jacs.4c00451.

35. Jiang, A.Y., Witten, J., Raji, I.O., Eweje, F., MacIsaac, C., Meng, S., Oladimeji, F.A., Hu, Y., Manan, R.S., Langer, R., and Anderson, D.G. (2024). Combinatorial development of nebulized mRNA delivery formulations for the lungs. Nat. Nanotechnol. *19*, 364–375. https://doi.org/10.1038/s41565-023-01548-3.

36. Oliveira, G.S., Oliveira, M.L.S., Miyaji, E.N., and Rodrigues, T.C. (2021). Pneumococcal Vaccines: Past Findings, Present Work, and Future Strategies. Vaccines (Basel) *9*, 1338. https://doi.org/10.3390/vaccines9111338.

37. Pichichero, M.E., Khan, M.N., and Xu, Q. (2016). Next generation protein based Streptococcus pneumoniae vaccines. Hum. Vaccines Immunother. *12*, 194–205. https://doi.org/10.1080/21645515.2015.1052198.

38. Nitika, Wei, J., Wei, J., and Hui, A.M. (2021). The Development of mRNA Vaccines for Infectious Diseases: Recent Updates. Infect. Drug Resist. *14*, 5271–5285. https://doi.org/10.2147/IDR.S341694.

39. Ozdilek, A., Paschall, A.V., Dookwah, M., Tiemeyer, M., and Avci, F.Y. (2020). Host protein glycosylation in nucleic acid vaccines as a potential hurdle in vaccine design for nonviral pathogens. Proc. Natl. Acad. Sci. USA *117*, 1280–1282. https://doi.org/10.1073/pnas.1916131117.

40. Anderson, R., Nel, J.G., and Feldman, C. (2018). Multifaceted Role of Pneumolysin in the Pathogenesis of Myocardial Injury in Community-Acquired Pneumonia. Int. J. Mol. Sci. *19*, 1147. https://doi.org/10.3390/ijms19041147.

41. Chen, H., Ren, X., Xu, S., Zhang, D., and Han, T. (2022). Optimization of Lipid Nanoformulations for Effective mRNA Delivery. Int. J. Nanomed. *17*, 2893–2905. https://doi.org/10.2147/IJN.S363990.

OMTN, Volume *36*

Supplemental information

Development of a novel-ionizable-lipid-based mRNA vaccine for broad protection against *Streptococcus pneumoniae*

Shi Xu, Guoqing Qi, Rui Liu, Shang Liu, Aili Wang, Wei Li, Keyue Ruan, Lingzhi Zhan, Lianshun Wang, Caiyi Fei, Jiyang Zhao, Xue Zhang, Qin Yu, Mengwei Xu, Jing Li, and Tiyun Han

Supplemental Information

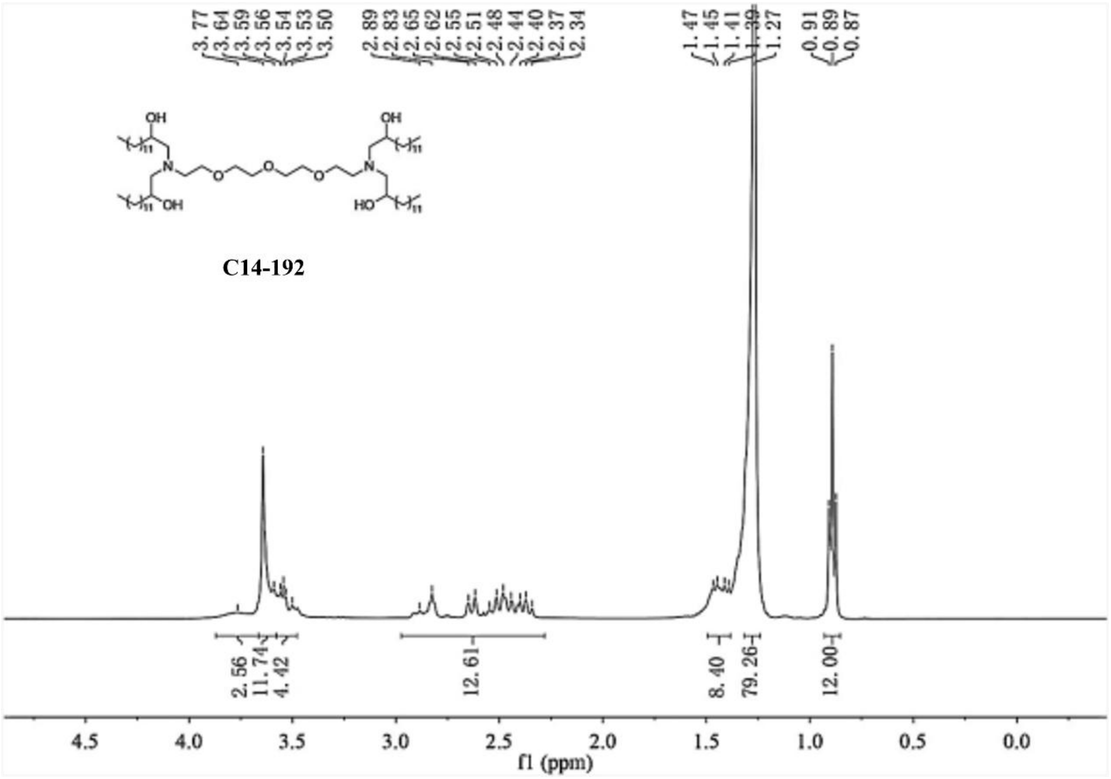

Figure S1. 1H nuclear magnetic resonance (1H NMR) spectra analysis of synthesized C14-192 cationic lipid compound.

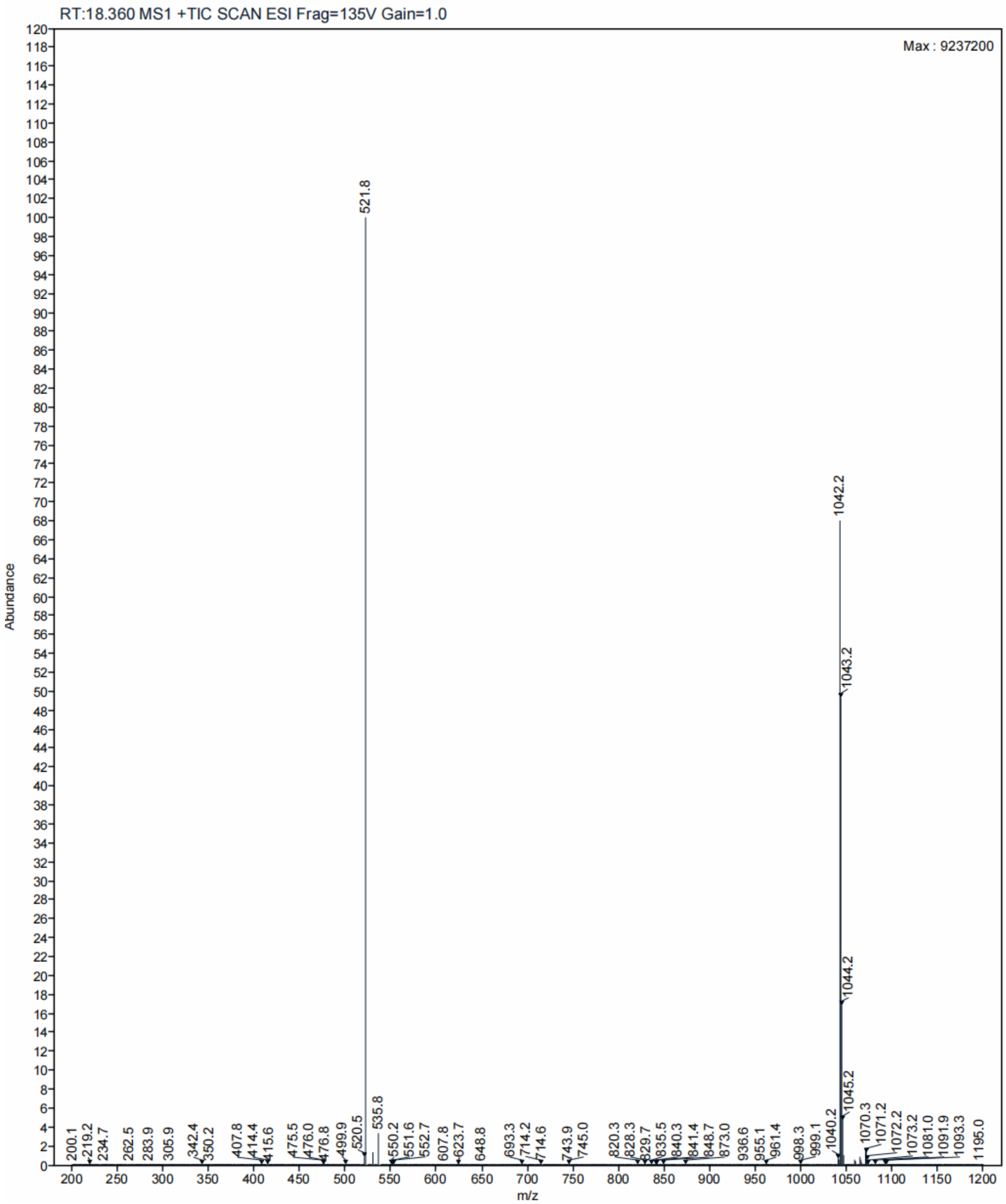

Figure S2. Liquid chromatography-mass spectrometry (LC-MS) analysis of synthesized C14-192 cationic lipid compound.

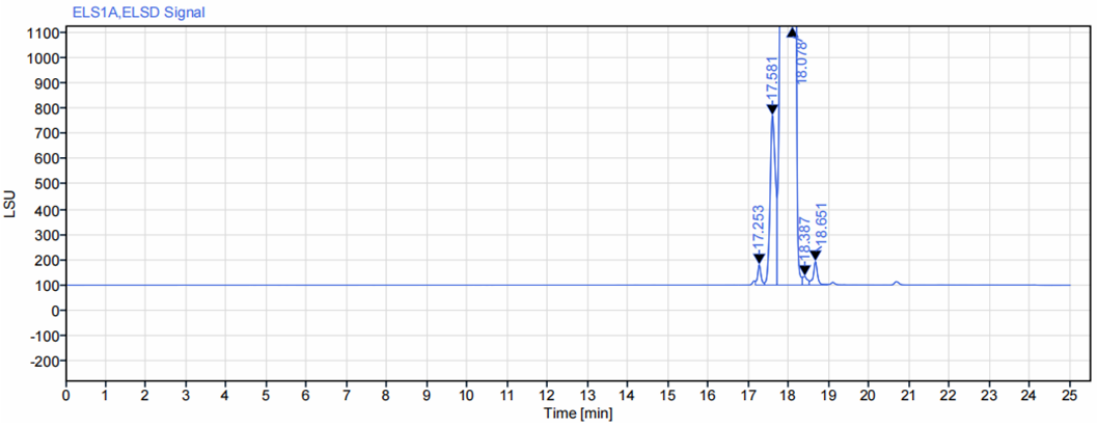

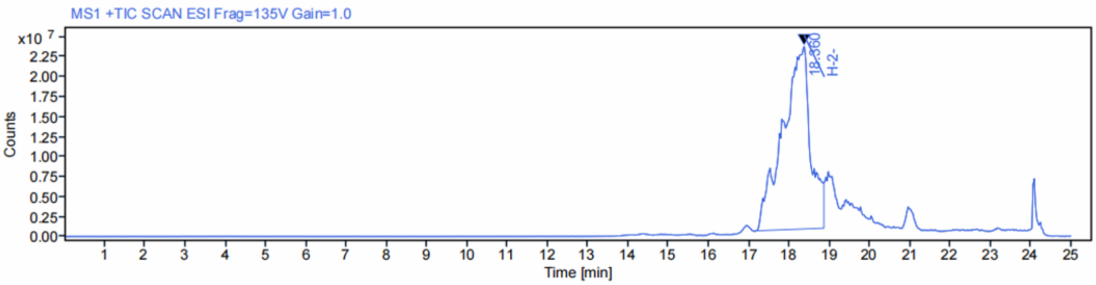

Signal: ELS1A,ELSD Signal

Name	RT [min]	RRT	Area	Height	Area%	TF	Plates	S/N	Rs.
	17.253	0.95	465.043	78.80126	0.107	1.2	243234	3,574	
	17.581	0.97	6188.606	667.96301	1.430	0.9	56705	30,299	1.480<br>02
H-2	18.078	1.00	425316.972	39229.175<br>68	98.245	0.7	66541	1,779,4<br>21	1.707<br>93
	18.387	1.02	271.798	33.44834	0.063	1.4	70731	1,517	0.840<br>90
	18.651	1.03	673.216	92.02648	0.156	1.0	218212	4,174	0.840<br>13
		Sum	432915.6339						

Signal: MS1 +TIC SCAN ESI Frag=135V Gain=1.0

Name	RT [min]	RRT	Area	Height	Area%	TF	Plates	S/N	Rs.
H-2-	18.360	1.00	1095383858.<br>134	22948336.1<br>5574	100.000	0.7	3467	575	
		Sum	1095383858.<br>1335						

Figure S3. High-performance liquid chromatography (HPLC) analysis of synthesized C14-192 cationic lipid compound.

a

	LNP (molar ratio)	mRNA	Diameter (nm) ± SD	PDI	ZP (mV)	Encapsulation Efficiency
1	SM102 : DSPC : Cholesterol : DMG-PEG 2000 = 50 : 10 : 38.5 : 1.5	Luciferase	119.84 ± 39.03	0.102	3.33	93%
2	C14-192 : DSPC : Cholesterol : DMG-PEG 2000 = 50 : 10 : 38.5 : 1.5	Luciferase	130.44 ± 43.15	0.126	1.20	91%
3	C14-192 : DSPC : Cholesterol : DMG-PEG 2000 = 45 : 8 : 45.5 : 2	Luciferase	131.07 ± 53.47	0.118	1.37	90%
4	C14-192 : DSPC : β-Sitosterol : DMG-PEG 2000 = 45 : 8 : 45.5 : 2	Luciferase	133.06 ± 53.08	0.175	1.80	90%

b

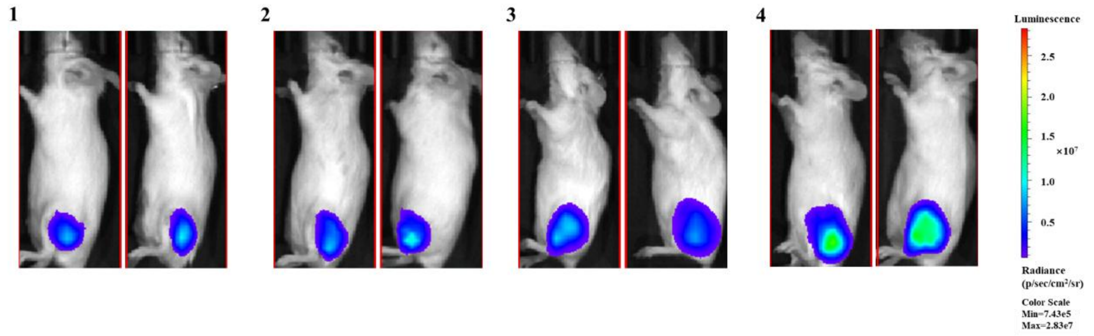

Figure S4. Characterization and analysis of the in vivo delivery efficiency of lipid nanoparticles (LNPs) with different components or molar ratios. (a) Characterization of four LNPs with varying lipid components or molar ratios, including size distribution, polydispersity index (PDI), zeta potential (ZP), and encapsulation efficiency. (b) Fluorescence intensity image of mice injected with the indicated luciferase mRNA-LNPs for 24 hours. Representative results are presented as means ± standard deviation (SD).

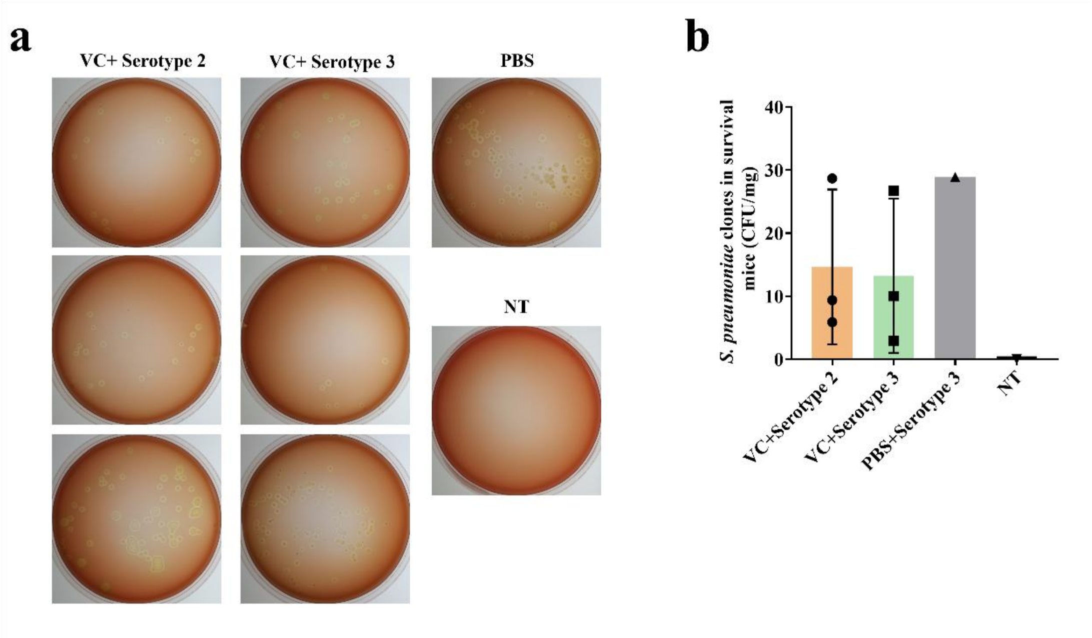

Figure S5. Bacterial load assay for lung tissue from representative survival mice pre-vaccinated with Vaccine C (VC) and infected with lethal dose of *S. pneumoniae* serotype 2 or serotype3, showing (a) the representative captures and (b) the average number of bacterial clones in Columbia blood agar plates. Representative results are shown as mean ± SD.
